# The effect of physical activity on white matter integrity in aging and prodromal to mild Alzheimer’s disease with vascular comorbidity

**DOI:** 10.3389/fnagi.2023.1096798

**Published:** 2023-06-21

**Authors:** Srijan Konwar, Riccardo Manca, Matteo De Marco, Hilkka Soininen, Annalena Venneri

**Affiliations:** ^1^Department of Life Sciences, Brunel University London, Uxbridge, United Kingdom; ^2^Department of Medicine and Surgery, University of Parma, Parma, Italy; ^3^Department of Neurology, University of Eastern Finland, Kuopio, Finland

**Keywords:** physical activity, white matter integrity, vascular burden, DTI, Alzheimer’s disease

## Abstract

**Background:**

Physical activity is a modifiable lifestyle factor that has been previously associated with reduced vascular burden and reduced risk of dementia.

**Objectives:**

This study tested whether physical activity (i.e., being inactive vs. active) contributed to preservation of white matter microstructure in healthy aging controls and patients in prodromal to mild Alzheimer’s disease with low/high vascular burden.

**Materials:**

A total of 213 participants were recruited from memory clinics. They were classified as being either physically active (*n* = 113) or inactive (*n* = 100) based on the Cardiovascular Risk Factors, Aging and Dementia (CAIDE) questionnaire. Diffusion-weighted images were acquired for all participants and pre-processed based on a standard protocol.

**Methods:**

A factorial design using voxel-wise tract-based spatial statistics (TBSS) was adopted, with 5,000 permutations and threshold-free cluster enhancement (TFCE), to identify significant clusters for fractional anisotropy (FA), axial diffusivity (AxD), mean diffusivity (MD), and radial diffusivity (RD).

**Results:**

Clusters of higher FA and lower AxD, MD, and RD values were found for physically active compared with inactive participants that were widespread covering mainly association and projection tracts but also some commissural tracts. A three-way Group × Physical Activity × Vascular Burden interaction effect was found for FA mostly in a variety of projection tracts with a right predominance, and some commissural and association tracts. *Post hoc* analyses revealed higher FA in patients with high vascular burden who were physically active compared with those patients with high vascular burden who were inactive mainly in projection and association/limbic tracts with a right predominance. Additionally, higher FA was observed in physically active patients with high vascular burden as compared with physically inactive controls with high vascular burden, mainly in bilateral projection fibers and cerebellar regions.

**Conclusion:**

Voxel-wise TBSS analysis revealed better preservation of white matter microstructure that was prominent in the high-risk group such as the patients with high vascular burden, specifically those who were physically active. The beneficial effects of physical activity on white matter microstructure were not observed in the controls.

## 1. Introduction

An early review of longitudinal data on lifestyle factors found that maintaining social engagement and physical activity might reduce the risk of dementia (Fratiglioni et al., 2004). The beneficial impact of physical activity on dementia risk has been observed in both men and women (Ihira et al., 2022). In line with the findings above, an updated Lancet commission report has listed physical inactivity as one of the 12 possible modifiable risk factors that, altogether, account for about 40% of all-cause dementia cases (Livingston et al., 2020).

The protective role of physical activity against cognitive decline is likely to be mediated by brain health preservation. In fact, some of the early intervention studies looking at the effects of cardiovascular fitness on cognitively normal older adults have found that aerobic exercise significantly increases both gray and white matter (WM) volume in prefrontal and temporal regions compared with non-aerobic exercise (Colcombe et al., 2006).

In recent years, Diffusion Tensor Imaging (DTI) has become a popular method to probe WM microstructure by measuring the diffusion of water molecules (O’Donnell and Westin, 2011). In their one-year longitudinal study, Voss et al. (2012) did not find a significant main effect of exercise condition (walking or stretching) on WM microstructure in any of the regions of interests (ROI) investigated. The only significant difference was an increase in fractional anisotropy (FA) in the prefrontal areas between exercise conditions. *Post hoc* within-group analyses showed that aerobic fitness was positively correlated with increased FA in prefrontal, parietal and temporal areas in the walking condition only. Similarly, cognitive performance was not different between conditions, and increased aerobic fitness resulted in better performance on the Digit Span test (backward condition) in the walking group only. A study by Perea et al. (2015) looked at the relationship between cardiorespiratory fitness and WM integrity in older adults with early AD, and found a positive association between peak oxygen consumption (VO2 peak) and FA in the right inferior fronto-occipital fasciculus (IFOF) and cingulum, although these results emerged only when no correction for multiple comparisons was applied. Physical activity has also been found significantly associated with lower mean diffusivity (MD) in the middle temporal lobe, and cingulate cortex in a sample of community-dwelling older adults (Tian et al., 2014). However, the study failed to show any association between physical activity and other neuroimaging markers such as FA and WM hyperintensities (WMH). Despite these contradictory results, a very recent systematic review found that the most well documented findings suggest that physical activity tends to have beneficial effects (lower atrophy) in specific areas such as hippocampal, temporal and frontal areas in cognitively healthy older adults who are genetically at risk for dementia (Domingos et al., 2021). In general, high FA reflects an undamaged axon that is well myelinated, whereas low FA is an indication of WM injury and poor integrity (Nir et al., 2013). MD is sensitive to tissue alterations such that any damage to WM (e.g., loss of myelin) results in MD increases (Nir et al., 2013), and can be affected by structural changes such as edema or inflammation (Alexander et al., 2007). In mouse models of transient optic nerve ischemia, significantly lower axial diffusivity (AxD) values were found post-injury, suggesting that this parameter is an indicator of axonal injury, typically accompanied by radial diffusivity (RD) increases, with this latter parameter indicating demyelination (Song et al., 2003). Although patterns of AxD alterations in AD do not always follow the same trend (e.g., Huang and Auchus, 2007), the general pattern observed in this disease consists of FA reductions and increases in AxD, MD, and RD (Mayo et al., 2019).

Pure cases of AD seldom are seen in the clinical population, and AD-related neuropathological changes often coexist with cerebrovascular alterations (Kivipelto et al., 2001). While individual vascular risk factors are associated with a higher risk of AD, the cumulative presence of multiple vascular risk factors is associated with a greater risk of probable or possible AD (Luchsinger et al., 2005). In a sample of cognitively healthy older adults, Maillard et al. (2015) found that, while exposure to a single vascular risk factor was associated with reduced FA in relatively small regions, FA values were not much different than those observed in participants with no vascular risks. However, those with 2 or more vascular risk factors had widespread WM microstructural damage, particularly in the splenium of the corpus callosum (CC) and thalamic radiation tracts (Maillard et al., 2015).

Despite a growing interest in the beneficial effects of physical activity and of an active lifestyle, few studies have investigated how varying degree of physical activity modulates any brain structural and functional changes in patients across the dementia spectrum, with coexisting vascular comorbidities. Various studies have examined the relationship between cardiorespiratory fitness and neurostructural integrity (brain volume, cortical thickness and WM microstructure), revealing a positive association between VO2 peak/VO2 max (maximum oxygen consumption) and FA in middle-aged adults (~54 ± 4 years) trained with aerobic exercise (Tarumi et al., 2020), healthy elderly adults (Oberlin et al., 2016), and amnestic mild cognitive impairment (MCI) patients (Ding et al., 2017), with this pattern stretching across limbic and long-range association tracts.

Another investigation examined the effect on FA of varying regimes of physical activity (light, moderate and vigorous) as well as VO2 max in sedentary, cognitively unimpaired older adults. The reported outcome was that higher physical activity and cardiorespiratory fitness correlated with higher FA (Burzynska et al., 2014). A further study focused on octogenarians with a sedentary lifestyle and a history of cardiometabolic conditions (i.e., diabetes and stroke): a positive association was found between aspects of physical activity measured over 7 consecutive days (active energy expenditure, number of steps walked and time spent exercising) and FA, and cognitive status (Tian et al., 2015).

Little is known about what role physical activity plays in modulating the relationship between multiple cardiovascular risk factors and WM microstructure in patients with a clinical diagnosis of either MCI or mild dementia due to AD. Therefore, the goal of this study was to investigate the effects of physical activity on WM microstructure in cognitively healthy controls and patients with MCI/dementia due to AD with either low or high vascular burden. We hypothesized that physically active participants would present with higher FA, lower AxD, MD, and RD and that the effect of physical activity would be more prominent among those with the greatest vascular risk, i.e., controls and patients with high vascular burden. We hypothesized that participants with high vascular burden would have pre-existing microstructural damage that would be greater than in those with low burden and, based on this assumption, physical activity would, most likely, be beneficial to participants in this condition than in those with low burden.

## 2. Materials and methods

### 2.1. Participants

A total of 213 participants were recruited as part of the EU-funded Virtual Physiological Human: Dementia Research Enabled by IT (VPH-DARE@IT) initiative. The aim of the VPH-DARE@IT project was to develop a comprehensive modeling approach to support and improve diagnosis and prognosis of dementia by collecting and analyzing a wealth of data including cognitive performance, multimodal MRI brain images, biological samples and lifestyle factors. The current study was funded by the European Union Seventh Framework Programme (FP7/2007–2013)—under Grant Agreement no. 601055 VPH-DARE@IT.

Recruitment was carried out in secondary-care clinical settings (a Department of Clinical Neurology), by teams of specialists that included a consultant neurologist, a clinical neuropsychologist and a neuroradiologist. As neurology referrals are typically sorted according to the sub-specialty that best suits their profile of symptoms (i.e., epilepsy, neuromuscular disorders, multiple sclerosis, etc.) potential candidates referred to the site’s memory clinic were systematically scrutinized for inclusion. Prior to selection, an extensive clinical examination was conducted to screen participants eligible for the study. AD-dementia patients were diagnosed based on the criteria from the National Institute of Neurological and Communicative Disorders and Stroke Alzheimer’s Disease and Related Disorders Association (NINCDS-ADRDA) (McKhann et al., 2011). Participants with MCI due to AD were diagnosed based on guidelines by the National Institute on Aging and the Alzheimer’s Association (Albert et al., 2011). Patients’ clinical diagnoses were supported by either cerebrospinal fluid biomarkers or plasma biomarkers for a proportion of the sample and clinical follow up for at least 6 years for the remaining participants. Exclusion criteria were defined as follows: any medical or neurological conditions such as traumatic brain injury that can cause cognitive impairment; any abnormality in MRI images other than those expected in aging or neurodegeneration; radiological evidence of acute or chronic cerebrovascular disease; presence/history of transient ischemic attacks; cardiovascular disease or presence of cardiovascular symptoms that are not under pharmacological control; uncontrolled seizures; peptic ulcer; folate or vitamin B12 deficiency; reduced serum thyroid-stimulating hormone; a heavy and prolonged medication regimen with treatments that could have a psychotropic effect; chronic psychiatric or psychological symptoms of severe intensity; presence of devices (such as pacemakers) incompatible with MRI scanning. The definition of this list of criteria, agreed among all recruitment sites, had the purpose of identifying prospective study candidates who were not affected by any other medical condition to a clinically-relevant degree. Additionally, all patients with a form of dementia were excluded if they had a Clinical Dementia Rating score > 1. This was carried out to make sure that individuals with a clinical diagnosis of dementia were at a mild stage. MRI and the rest of the clinical and behavioral examinations were carried out, on average, within a two-week period. Control participants were recruited from opportunity sampling including patients’ spouses and people who had heard about the study via word of mouth. All controls completed the same consensus-based diagnostic procedures as the group of clinically-established patients.

The overall sample consisted of a total of 213 participants that included 77 controls, 89 MCI and 47 cases of AD dementia. As cases of MCI and dementia in the current study both fall under the same disease continuum, they were combined to form a unique patient group (*n* = 136).

All experimental procedures were carried out according to the guidelines of the Declaration of Helsinki, and written informed consent was obtained from all participants. Ethical approval for the research was obtained from the Regional Ethics Committee of Yorkshire and Humber (Ref No: 12/YH/0474) for patients recruited at the memory clinic in Sheffield, United Kingdom, and from the ethics committee of the Northern Savonia Hospital District for the participants recruited from the memory clinic in Kuopio, Finland.

### 2.2. Physical activity

Participants were classified as being either physically active (*n* = 113) or inactive (*n* = 100) based on a questionnaire that is part of the Cardiovascular Risk Factors, Aging and Dementia (CAIDE) risk score, an instrument validated to be used among cognitively healthy adults (Stephen et al., 2021) and adults with MCI (Rosenberg et al., 2019) to estimate late-life risk of dementia. To be considered as active, participants must have engaged in physical activity causing sweating and breathlessness at least twice a week for 20–30 min (Kivipelto et al., 2006). Participants were asked the following question: How often do you exercise for at least 20 min so that you at least are mildly out of breath and sweaty? Responses were coded as (1) 5 times a week or more often, (2) 4 times a week, (3) 3 times a week, (4) twice a week, (5) once a week, (6) less than once a week, (7) I have a disability or disease which does not enable me to exercise. Participants accompanied by a carer had the opportunity to receive their support in completing the questionnaire.

### 2.3. Vascular burden

To quantify vascular burden, we utilized the office-based Framingham risk score, a version of the Framingham General Cardiovascular Risk Profile (D’Agostino et al., 2008) that is a gender-specific risk algorithm that provides an overall estimate of having cardiovascular disease based on the following variables: age, systolic blood pressure (treated or untreated), smoking, diabetes and body-mass index. The Framingham risk score can be classified as: (1) low—less than 10%, (2) medium—between 10 and 20%, and (3) high—above 20%. In this sample, the number of low-risk participants was very low and, hence, it was decided to combine participants with low and medium risk scores in a “low-burden” group (*n* = 78), whereas participants with high risk scores were classified as the “high-burden” group (*n* = 135).

### 2.4. MRI image acquisition

Diffusion-weighted images were acquired with 32 directions and the following specifications: voxel size: 2.5 mm × 2.5 mm × 2.5 mm, repetition time 3 s, echo delay time 98 ms, flip angle 90°, field of view 240 × 120 × 240 mm, matrix size 96 × 94 mm^2^. Neuroanatomical T1-weighted (T1W) Turbo Field Echo images were acquired with a Philips Ingenia 3 T scanner with the following parameters: Voxel size: 0.94 x 0.94 x 1.00 mm^3^; repetition time 8.2 ms; echo delay time 3.8 ms; flip angle 8; field of view 256 mm; matrix size 256 x 256 x 170.

### 2.5. Imaging analysis

The DTI images were processed using the Functional MRI of the Brain Software Library (FSL) version 6.0.4 (Smith et al., 2004). Correction for eddy currents and motion artifacts was performed on the raw diffusion weighted images by aligning them to the b0-volume image using the eddy_correct command in FSL. This was followed by the removal of non-brain structures using the Brain Extraction Tool (Smith, 2002). A fractional intensity threshold of 0.5 was applied to the brain image and the binary brain mask image. The diffusion tensor was created using the FMRIB Diffusion Toolbox (DTIFIT program) of FSL to obtain FA, AxD, MD and RD images. Quantification of global tissue map volumes was carried out on T1W images with the get_totals script (http://www0.cs.ucl.ac.uk/staff/g.ridgway/vbm/get_totalsm) to calculate total intracranial volumes (TIV) and thus account for overall head size variability.

### 2.6. Pre-processing

Voxel-wise non-linear registration to the standard MNI FMRIB58_FA template was carried out to normalize the FA images. After this alignment, FA images were averaged to create a mean FA skeleton mask that reflected the center of all tracts common to the entire sample. After this, all the FA images were projected onto the skeleton mask and a threshold of 0.2 was applied to exclude any voxels located in gray matter or cerebrospinal fluid. Similar procedures were also followed for AxD, MD and RD images, whereby the output images were projected onto the mean FA skeleton mask.

### 2.7. Statistical analyses

#### 2.7.1. Descriptive statistics

All demographic variables were analyzed using IBM SPSS Statistics, version 28 (IBM Corp., Armonk, NY, United States). Continuous variables underwent preliminary checks to assess normality of data distribution and were compared across conditions using the Kruskal-Wallis test, for non-normally distributed variables, and One-Way Analysis of Variance (ANOVA), for normally distributed variables. All categorical variables were compared across groups using Pearson Chi-Square test (χ^2^). The significance threshold was set at *p* < 0.05 (two-tailed). *Post hoc* analyses were carried out between groups using pair-wise comparisons adjusted for multiple comparisons (Bonferroni correction).

#### 2.7.2. Voxel-wise analyses

Voxel-wise statistical analyses were carried out using Tract-based spatial statistics (TBSS) (Smith et al., 2006) to investigate significant differences in FA, AxD, MD, and RD, across diagnoses (controls vs. patients), physical activity (active vs. inactive) and vascular burden (low burden vs. high burden) groups. Whole-brain voxel-wise analyses were carried out using a non-parametric method based on permutations (Winkler et al., 2014). We used FSL’s *randomise* command with 5,000 permutations and threshold-free cluster enhancement (TFCE) to identify significant clusters (Smith and Nichols, 2009). For all analyses, site of recruitment, TIV and years of education were used as covariates. Site of recruitment, a categorical covariate, was included in the models to regress out that proportion of WM variability overtly or covertly dependent on aspects intrinsic to the country of origin (such as educational styles, dietary habits, outdoor temperatures or daylight periods) that could be, indirectly, at the basis of marginal aspects of neural trajectories. Age was not included as a covariate because age was already accounted for in the Framingham formula when computing the vascular scores. A factorial design was set up to examine the main effects of diagnostic group, physical activity and vascular burden and the various interaction effects among the 3 factors using a family-wise error corrected threshold (FWE *p* < 0.05). All clusters that survived the significant threshold corrected for multiple comparisons were labeled using the John Hopkins University (JHU) atlases, ICBM-DTI-81 white-matter labels atlas (Mori et al., 2008) and JHU white-matter tractography atlas (Wakana et al., 2007). Any regions left unclassified or undefined by the JHU atlases were labeled using the Talairach Daemon atlas (Lancaster et al., 2000).

For significant interaction effects, separate individual *post hoc* analyses were carried out between the different groups. Our main aim was to look at the effects of physical activity (inactive vs. active) within controls and patients with either low or high vascular burden. *Post hoc* comparisons were as follows:

1. Controls Active Low Burden vs. Controls Inactive Low Burden.
2. Controls Active High Burden vs. Controls Inactive High Burden.
3. Patients Active Low Burden vs. Patients Inactive Low Burden.
4. Patients Active High Burden vs. Patients Inactive High Burden.

A Bonferroni correction was applied to the FWE-corrected *p* for all 4 contrasts (*p* = 0.05/4 = 0.0125). A separate *post hoc* comparison between physically active patients with high vascular burden and inactive controls with high vascular burden was explored using an FWE-corrected *p* < 0.05 threshold. Differently from the above list, this fifth exploratory contrast tested for cross-diagnostic group differences between active and inactive participants and, for this reason, was thresholded at a more lenient cluster-forming *p*-value.

The entire set of inferential models was re-run including age, body-mass index, systolic blood pressure, smoking status and diabetes as covariates to provide statistical control for other potential sources of variability.

## 3. Results

### 3.1. Demographics

Groups differed significantly in age, apolipoprotein E (APOE) status, education, gray matter volume, Mini Mental State Examination (MMSE), sex, TIV, vascular scores, WM volume (see Table 1). Additionally, demographic variables in relation to each of the three binary predictors (i.e., “controls vs. patients,” “active vs. inactive,” “low burden vs. high burden”) are reported in Supplementary Tables S1–S3. Differences between controls and patients were found, in the expected direction, for variables known to be implicated in cognitive impairment, such as years of education, global gray-matter volumes and Mini Mental State Examination scores, but the two sub-cohorts did not differ in terms of vascular burden or vascular risk factors (Supplementary Table S2).

**Table 1:** Demographic and neural characteristics of the sample.

Variables	Whole cohort (*n* = 213)	Inactive controls low burden (*n* = 10)	Inactive controls high burden (*n* = 22)	Active controls low burden (*n* = 19)	Active controls high burden (*n* = 26)	Inactive patients low burden (*n* = 22)	Inactive patients high burden (*n* = 46)	Active patients low burden (*n* = 27)	Active patients high burden (*n* = 41)	*p*
Demographic/clinical										
Age (years)^a,b^	69 (17)	61.50 (17)	73 (9)	58 (14)	71.50 (12)	62 (15)	78 (10)	59 (17)	69 (13)▼^***^	**<0.001**
APOE status (No. of Ɛ4 carriers/No. of individuals with APOE data, %)^c,d^	77/193 (39.90%)	2/9 (22.22%)	1/17 (5.88%)	7/19 (36.84%)	4/22 (18.18%)	8/21 (38.10%)	20/39 (51.28%)	15/27 (55.56%)	20/39 (51.28%)^♦**^	**0.004**
BMI^e,f^	26.61 (4.73)	24.76 (6.49)	25.39 (5.63)	27.18 (6.57)	28.15 (4.72)	26.16 (10.38)	25.81 (5.37)	24.09 (6.29)	26.47 (4.38)	0.054
Education (years)^a,b^	12 (6)	16.50 (9)	12.50 (7)	15 (3)	13 (6)	11 (2)	10 (4)	14 (5)▲^*^	9 (7)^♦**^	**<0.001**
MMSE^a,b^	27 (5)	29 (4)	28.50 (3)	29 (2)	28.50 (1)	23.50 (5)	25.50 (5)	27 (6)▲^*^	26 (4)^♦***^	**<0.001**
Sex (Females %)^c,d^	117 (54.93%)	7 (70%)	15 (68.18%)	15 (78.95%)	12 (46.15%)	14 (63.64%)	26 (56.52%)	18 (66.67%)	10 (24.39%)▼^**,♦**^	**0.001**
SBP^a,b^	142 (27)	124 (25)	150 (30)	141 (23)	145.50 (21)	123.50 (14)	149.50 (32)	137 (17)▲^**^	149 (24)	**<0.001**
Vascular scores^e,f^	30.26 (19.05)	16.32 (13.09)	38.32 (32.23)	13.76 (6.76)	30.54 (11.76)	13.13 (5.93)	43.61 (25.88)	14.65 (6.72)	31.31 (22.27)▼^**^	**<0.001**
Neuroimaging										
GMV (mL)^e,f^	599.47 (74.55)	634.33 (74.35)	586.51 (38.28)	669.59 (65.39)	616.71 (63.00)^ 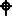 *^	593.87 (69.07)	543.47 (64.92)	624.00 (73.49)	604.19 (72.81)▼^***^	**<0.001**
WMV (mL)^e,f^	404.23 (55.75)	378.72 (94.41)	379.34 (52.24)	427.17 (61.63)^†*^	424.53 (74.15)^ 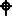 **^	393.82 (61.46)	376.13 (75.70)	407.30 (67.99)	415.59 (103.43)▼^*^	**0.001**
TIV (mL)^e,f^	1453.05 (154.37)	1366.47 (158.48)	1391.88 (130.10)	1494.76 (148.17)^†*^	1507.33 (180.44)^ 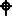 *^	1434.74 (162.35)	1430.83 (145.90)	1450.90 (127.23)	1489.44 (156.32)^♦*^	**0.037**

APOE, apolipoprotein E; BMI, body mass index; GMV, gray matter volume; MMSE, mini mental state examination; No, number; SBP, systolic blood pressure; TIV, total intracranial volume; WMV, white matter volume. ^a^Median (interquartile range); ^b^Kruskall-Wallis; ^c^Frequencies; ^d^Chi-Square test; ^e^Mean (standard deviation); ^f^One-Way Analysis of Variance. All significance levels are at *p* < 0.05; ^*^p < 0.05, ^**^p < 0.01, ^***^p < 0.001; ^†^Controls Active Low Burden vs. Controls Inactive Low Burden; ^
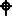
^Controls Active High Burden vs. Controls Inactive High Burden; ^▲^Patients Active Low Burden vs. Patients Inactive Low Burden; ^▼^Patients Active High Burden vs. Patients Inactive High Burden; ^♦^Patients Active High Burden vs. Controls Inactive High Burden. APOE data were only available for a proportion of the cohort; Values in bold indicate *p* values < 0.05.

### 3.2. Main effect of group and vascular burden on white matter microstructural integrity

A main effect of group revealed that controls had higher FA than patients mainly in left-sided association tracts, parietal and temporal areas (Supplementary Tables S4–S7). Whereas the patients had higher AxD, MD, and RD than the controls in widespread regions covering association, commissural and projection tracts. Moreover, the low vascular burden group had higher FA than the high vascular burden group mainly in the commissural and left projection tracts. The high vascular burden group had greater AxD and MD in mostly left projection tracts with higher RD in bilateral projection, association and commissural tracts (Supplementary Tables S8–S11).

### 3.3. Main effect of physical activity on white matter microstructural integrity

A significant main effect of physical activity was observed on FA across a widespread network of WM tracts: participants who were physically active had higher FA in tracts such as the right fornix and superior longitudinal fasciculus (SLF) temporal part and bilateral cingulum (Supplementary Table S12). By contrast, non-physically active participants were found to have higher AxD, MD, and RD (Supplementary Tables S13–S15). In particular, higher AxD was observed in the right retrolenticular part of the internal capsule, bilateral IFOF, left posterior thalamic radiation, left inferior longitudinal fasciculus (ILF), left sagittal stratum and left uncinate fasciculus. Higher MD values were observed in the tracts such as the left cingulum, left ILF, right SLF, bilateral retrolenticular part of internal capsule and left parietal lobe precuneal WM. Higher RD values, finally, were found primarily in the left cingulum, left ILF and left SLF.

### 3.4. Interaction effects between diagnostic group, physical activity, and vascular burden on white matter microstructural integrity

**Figure 1:**
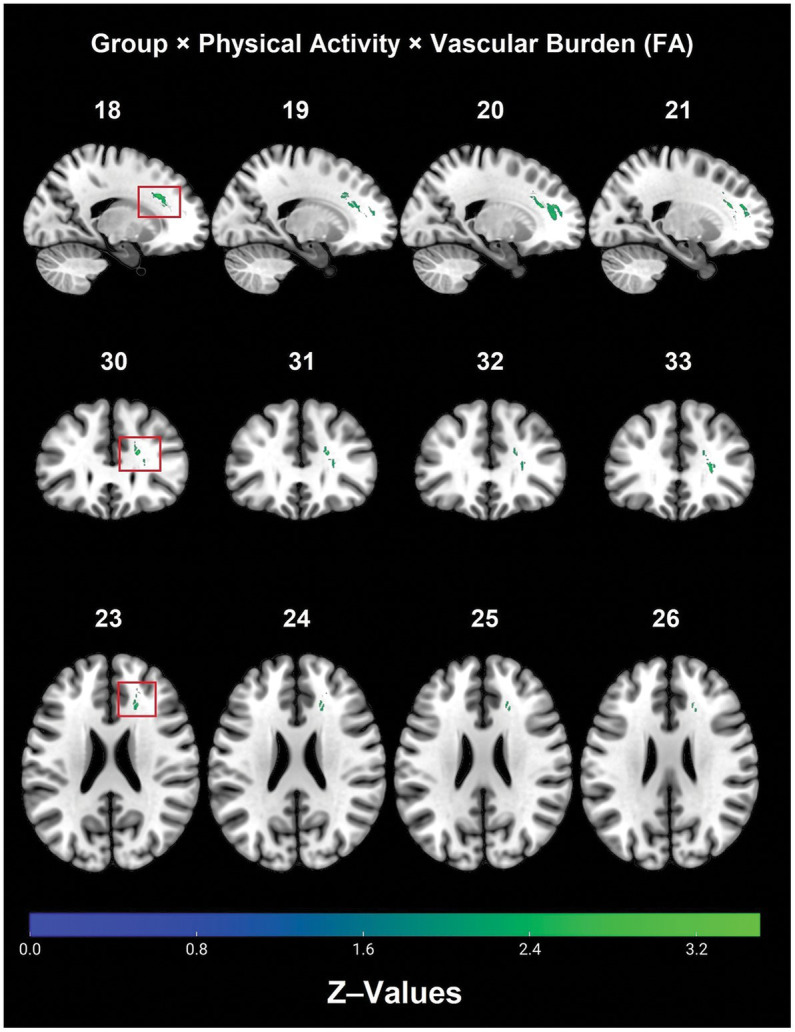
Significant diagnostic group × physical activity × vascular burden interaction effect on FA (TFCE, FWE, *p* < 0.05). FA, fractional anisotropy; FWE, family-wise error; TBSS, tract-based spatial statistics.

There was a significant 2 (Controls/Patients) × 2 (Active/Inactive) × 2 (Low Burden/High Burden) interaction effect observed for FA in WM tracts that included: right frontal lobe cingulate gyrus, right anterior corona radiata, forceps minor, right anterior thalamic radiation and right IFOF (see Table 2 and Figure 1).

**Table 2:** Interactive effects of diagnostic group × physical activity × vascular burden show differences in FA in these WM tracts and regions (FWE *p* < 0.05).

				MNI coordinates of local maxima			WM tracts		
Clusters	Voxels	Value	*p*-value	*X*	*Y*	*Z*	JHU-ICBM-DTI-81 WM labels	JHU-WM tractography atlas	Talairach Daemon
1	373	3.75	**0.047**	18	24	32	Unclassified		Right cerebrum, frontal lobe, cingulate gyrus, WM
		3.73	**0.047**	18	30	23	Right anterior corona radiata	Forceps minor	
		3.56	**0.047**	21	30	22	Right anterior corona radiata	Forceps minor	
		3.27	**0.047**	20	41	20	Unclassified	Forceps minor	
		3.23	**0.047**	20	43	11	Unclassified	Forceps minor	
		3.22	**0.047**	21	42	22	Unclassified	Right anterior thalamic radiation	
2	149	3.73	**0.048**	24	35	4	Right anterior corona radiata	Right inferior fronto-occipital fasciculus	
		3.65	**0.048**	24	34	8	Right anterior corona radiata	Right anterior thalamic radiation	
		3.63	**0.048**	23	35	6	Right anterior corona radiata	Right anterior thalamic radiation	
		3.5	**0.048**	27	35	8	Unclassified	Right inferior fronto-occipital fasciculus	
		3.39	**0.048**	25	37	12	Unclassified	Right anterior thalamic radiation	
		3.35	**0.048**	24	34	11	Right anterior corona radiata	Right anterior thalamic radiation	

Only clusters with > 100 voxels are listed; Talairach Daemon atlas was used when regions were either labeled as unclassified or undefined by JHU atlas; Values in bold indicate *p* values < 0.05.

**Figure 2:**
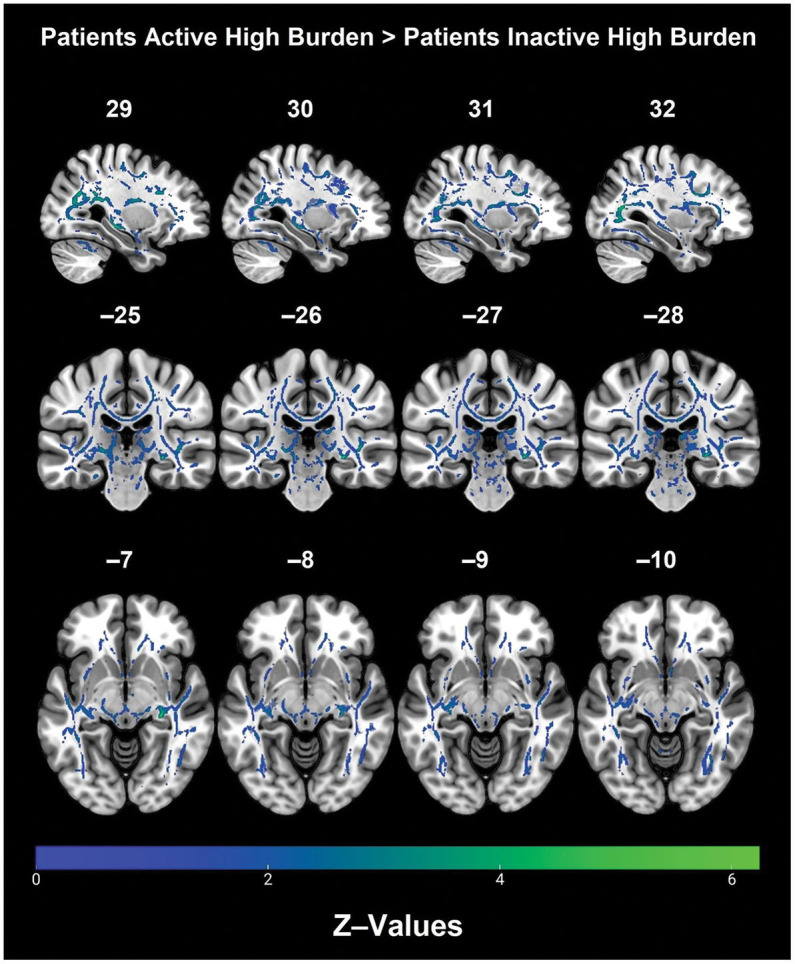
Regions with significantly higher FA in the physically active patients with high vascular burden as compared with non-physically active patients with high vascular burden. (TFCE). A Bonferroni correction was applied (*p* < 0.0125) to correct for multiple comparisons.

**Figure 3:**
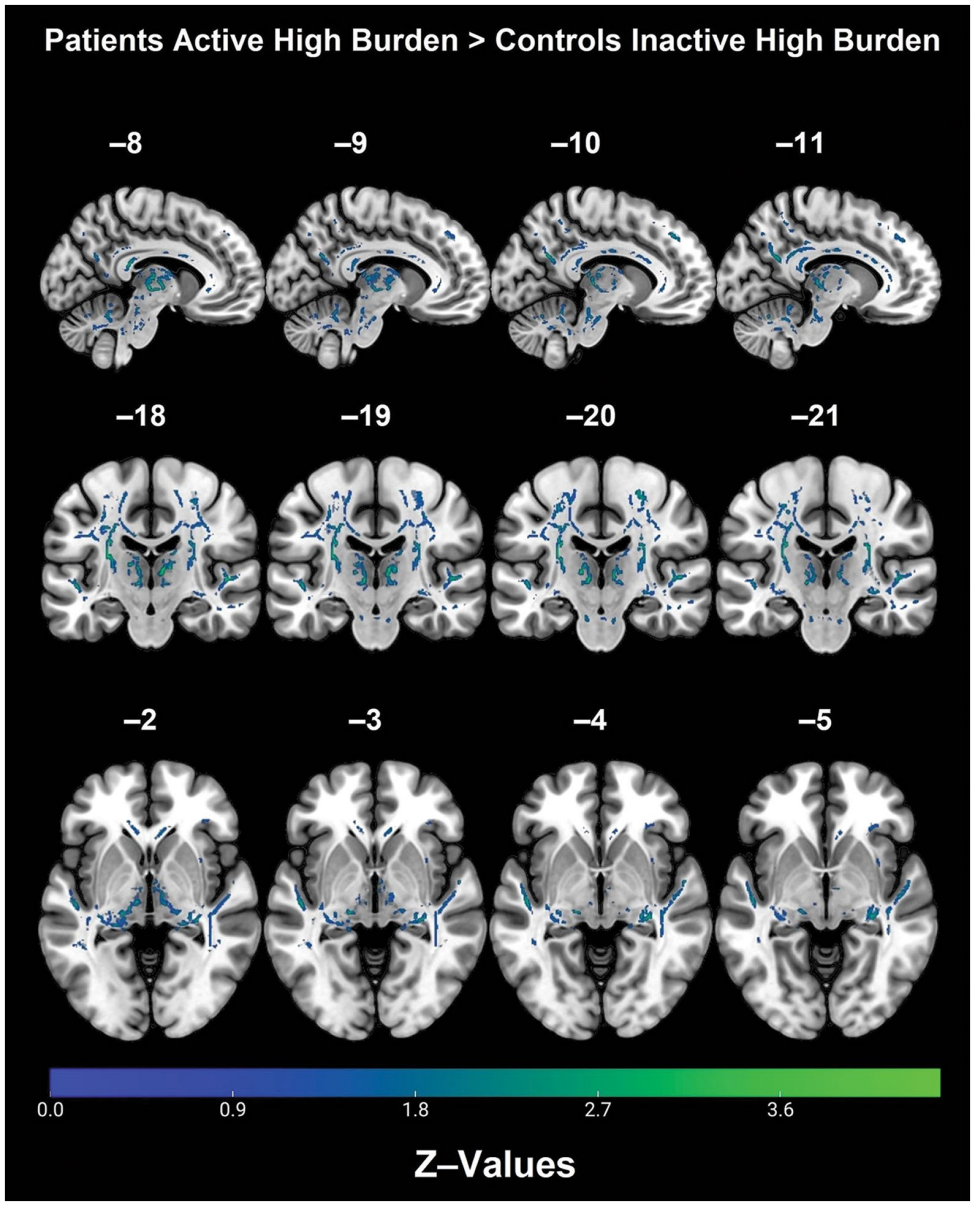
Regions with significantly higher FA in the physically active patients with high vascular burden as compared with non-physically active controls with high vascular burden (TFCE, FWE, *p* < 0.05).

*Post hoc* analyses within diagnostic groups revealed that physically active patients with high vascular burden had greater FA than non-physically active patients with high vascular burden. Specifically, higher FA values were observed in the right fornix (cres)/stria terminalis, right posterior thalamic radiation (including optic radiation), forceps major, right IFOF, right superior corona radiata, right SLF and right ILF (Table 3 and Figure 2). Furthermore, physically active patients with high vascular burden had higher FA than the non-physically active controls with high vascular burden. The regions that displayed higher FA were observed mainly in projection fibers and cerebellar areas (see Table 4 and Figure 3).

**Table 3:** *Post hoc* analyses show that patients with high vascular burden who were active had higher FA than those who were inactive in these WM tracts (FWE *p* < 0.0125).

				MNI coordinates of local maxima			WM tracts	
Clusters	Voxels	Value	*p-*value	*X*	*Y*	*Z*	JHU-ICBM-DTI-81 WM labels	JHU-WM tractography atlas
1	71,888	6.57	***p* < 0.001**	29	−25	−7	Right fornix (cres)/stria terminalis (cannot be resolved with current resolution)	
		5.9	***p* < 0.001**	32	−58	11	Right posterior thalamic radiation (include optic radiation)	Forceps major
		5.85	***p* < 0.001**	33	−67	18	Unclassified	Right inferior fronto-occipital fasciculus
		5.7	***p* < 0.001**	28	−8	23	Right superior corona radiata	Right superior longitudinal fasciculus
		5.7	***p* < 0.001**	33	−57	15	Right posterior thalamic radiation (include optic radiation)	Right inferior fronto-occipital fasciculus
		5.6	***p* < 0.001**	34	−60	17	Right posterior thalamic radiation (include optic radiation)	Right inferior longitudinal fasciculus

Bonferroni correction for multiple comparisons was applied (corrected *p* < 0.0125); Values in bold indicate *p* values < 0.0125.

**Table 4:** WM tracts with significantly higher FA values in physically active patients with high vascular burden compared with non-physically active controls with high vascular burden (FWE *p* < 0.05).

				MNI coordinates of local maxima			WM tracts		
Clusters	Voxels	Value	*p*-value	*X*	*Y*	*Z*	JHU-ICBM-DTI − 81 WM labels	JHU-WM tractography atlas	Talairach Daemon
1	15,916	4.85	**0.012**	−8	−18	−2	Unclassified	Left anterior thalamic radiation	
		4.73	**0.012**	−22	−8	14	Left posterior limb of internal capsule		
		4.65	**0.012**	−15	−26	13	Unclassified	Left anterior thalamic radiation	
		4.63	**0.012**	−29	−33	13	Left retrolenticular part of internal capsule		
		4.5	**0.012**	−12	−20	6	Unclassified	Left anterior thalamic radiation	
		4.41	**0.012**	−28	−18	17	Left external capsule	Left corticospinal tract	
2	12,725	5.03	**0.012**	27	−30	17	Right retrolenticular part of internal capsule		
		4.75	**0.012**	27	−26	15	Right retrolenticular part of internal capsule		
		4.66	**0.012**	50	0	−12	Unclassified	Right inferior longitudinal fasciculus	
		4.39	**0.012**	27	−28	18	Right retrolenticular part of internal capsule		
		4.3	**0.012**	27	−30	23	Right posterior corona radiata		
		4.2	**0.012**	31	−34	15	Right retrolenticular part of internal capsule		
3	3,085	4.43	**0.035**	8	−67	−21	Unclassified		Right cerebellum
		4.09	**0.035**	10	−42	−36	Right inferior cerebellar peduncle		
		3.92	**0.035**	−5	−69	−22	Unclassified		Left cerebellum
		3.9	**0.035**	24	−61	−20	Unclassified		Right cerebellum
		3.78	**0.035**	11	−71	−22	Unclassified		Right cerebellum
		3.73	**0.035**	24	−57	−22	Unclassified		Right cerebellum
4	2061	4.44	**0.034**	8	−9	5	Unclassified	Right anterior thalamic radiation	
		4.42	**0.034**	8	−18	8	Unclassified	Right anterior thalamic radiation	
		4.33	**0.034**	20	−30	0	Unclassified		Right cerebrum, sub-lobar, extra-nuclear, WM
		4.3	**0.034**	15	−16	8	Unclassified		Right cerebrum, sub-lobar, extra-nuclear, WM
		4.27	**0.034**	20	−28	4	Unclassified		Right cerebrum, sub-lobar, extra-nuclear, WM
		4.17	**0.034**	8	−19	4	Unclassified	Right anterior thalamic radiation	
5	886	4.41	**0.045**	8	−29	−32	Pontine crossing tract (a part of MCP)	Right corticospinal tract	
		4.29	**0.045**	6	−28	−33	Right corticospinal tract	Right corticospinal tract	
		4.22	**0.045**	8	−31	−35	Pontine crossing tract (a part of MCP)	Right corticospinal tract	
		4.03	**0.045**	8	−31	−33	Pontine crossing tract (a part of MCP)	Right corticospinal tract	
		3.77	**0.045**	7	−27	−30	Right corticospinal tract	Right corticospinal tract	
		3.39	**0.045**	5	−31	−32	Pontine crossing tract (a part of MCP)	Right corticospinal tract	

Only clusters with > 200 voxels are listed; Talairach Daemon atlas was used when regions were either labeled as unclassified or undefined by JHU atlas; MCP = middle cerebellar peduncle; Values in bold indicate *p* values < 0.05.

No significant effects were found in association with the contrasts going in the opposite direction as those described in this section.

To ascertain the robustness of the aforementioned pattern of findings, the entire set of inferential models was re-run with the inclusion of a further set of covariates (see Materials and methods section for details). The findings emerging from these additional models (reported in Supplementary Tables S16–S22) remained substantially unaltered.

## 4. Discussion

The findings of this study show a main effect of physical activity on WM microstructural integrity whereby participants who were physically active had higher FA than the inactive participants, mainly in right-lateralized association fibers, such as the right cingulum and right SLF. As expected, inactive participants had higher AxD, MD, and RD compared with the active participants. The main effect of physical activity on AxD and MD were mainly in the left association and projection fibers, whereas the main effect of physical activity on RD was mainly in the left limbic and association fibers. These findings expand on those by Tian et al. (2015) who showed that specific aspects of physical activity such as energy expenditure, step count and exercise duration were positively associated with FA in the map of normally-appearing WM (Tian et al., 2015). A higher step count was also associated with lower RD in this sample of oldest-old (i.e., > 80 years) sedentary adults with a history of cardiometabolic conditions (i.e., diabetes and stroke). A recent study found that physical activity as measured by physical activity energy expenditure in SuperAgers (i.e., older individuals with a memory performance comparable with or better than the average normative values expected of adults aged 45 years) was positively associated with FA in the genu and body of the CC, right SLF and forceps minor, in comparison with typical agers (Kim et al., 2020). The SuperAgers had also lower AxD in the CC, lower MD in the body and splenium of the CC, left SLF and ILF, and lower RD in the CC, left ILF and bilateral SLF (Kim et al., 2020). Along similar lines, a recent study carried out in a mixed sample of cognitively healthy adults and individuals with MCI found an association between actigraphy-estimated physical activity and both higher FA and lower RD (Soldan et al., 2022). This relationship was observed across multiple tracts, suggesting that the benefits of physical activity may be widespread and not localized to specific areas.

A significant three-way interaction between diagnosis, physical activity and vascular burden was also observed for FA mainly in right lateralized projection and commissural tracts. Within the patient group with high vascular burden, physical activity was associated with better preserved WM microstructure mainly in the right association fibers, as well as right projection fibers. Moreover, patients who were physically active with high vascular burden had greater FA than controls with high vascular burden who were inactive, primarily in right-lateralized projection fibers. No significant interactions were observed for AxD, MD, and RD.

These findings suggest that physical activity may contribute to preserving WM microstructure in patients harboring the greatest vascular risk. Consistently, MD is significantly influenced by the interplay of physical activity and age. In a study carried out in cognitively healthy older adults, the term modeling the interaction between age and the metric calculated by a triaxial accelerometer worn on the participants’ wrist was a significant predictor of MD across various white matter tracts (Wolf et al., 2020). Kim et al. (2020) provided complementary evidence by showing that physical activity is involved in the preservation of tracts (as shown by multiple TBSS metrics) that are implicated early in AD, such as the genu of the CC and association tracts like the ILF and SLF that are known to support memory functioning. Evidence linked to this beneficial effect was observed in areas such as association and projection fibers that have been deemed susceptible to aggregate vascular risk factors (Cox et al., 2019). These findings are in line with those of a review of DTI studies by Wassenaar et al. (2019) that found that modifiable risk factors, such as physical activity, are associated with higher FA values in various WM tracts, like commissural fibers such as the CC, corona radiata, fornix, internal capsule, cingulum, IFOF, SLF and uncinate fasciculus. Taken together, these and our findings support the view that physical activity tends to have an effect on WM microstructure at a global scale rather than at a localized level.

A previous study found that cognitively healthy older adults who were ε4 carriers and physically inactive had reduced hippocampal volumes compared with ε4 carriers who were physically active (Smith et al., 2014). Similar results were also obtained by Smith et al. (2011) who found that semantic memory processing as observed through brain activation patterns was most notable among ε4 carriers who were physically active. Although of different remit, these studies seem to suggest that the beneficial effect of physical activity may be more prominent among people at higher risk for dementia, such as ε4 carriers or people with high vascular burden.

Although beyond the scope of this paper, this study does not assess neuroinflammation that is known to accompany vascular burden (Hansson, 2005). Neuroinflammation has been observed in several neurodegenerative diseases and has also been known to contribute to the pathological dysfunction in AD (Calsolaro and Edison, 2016). Several cross-sectional studies have shown reduced inflammatory markers and C-reactive protein, Interleukin 6 (IL-6), specifically observed in active elderly as well as in patient populations such as MCI and AD, and Tumor necrosis factor α after exercise (Umegaki et al., 2021). Inflammation has been observed in MCI patients without any visible signs of amyloid deposition (Calsolaro and Edison, 2016). The role of DTI indices in explaining chronic inflammation with respect to physical activity modulation needs to be better clarified. In this study, we observed a pattern whereby the main effect of physical activity (Inactive > Active) was more pronounced for RD (significantly large cluster size of 72,872 voxels) than for AxD (cluster size of 25,047 voxels) and MD (cluster size of 25,047 voxels). These findings are important because some of the earliest studies have shown that initial damage to the optic nerve in mouse models can result in initial reduction in AxD during the early stages of demyelination whereas increases in RD is only observed when there is chronic demyelination (Winklewski et al., 2018). These findings from mouse models are in contradiction to DTI studies examining WM integrity in AD patients that found that AD patients had higher AxD and RD (Nir et al., 2013; Mayo et al., 2019). Future studies would need to examine the association between CSF/plasma inflammatory markers in AD and whether they have differential effects on DTI indices such as AxD and RD. Furthermore, it is important to understand the differential effects of physical activity on WM integrity stratified by amyloid status, tau status and various cardiovascular risk factors. Given that the main effect of physical activity was stronger for RD compared with AxD, this suggests that physical activity might be beneficial in preserving microstructure that is vulnerable to demyelination specifically the limbic association fibers, such as the cingulum (neurodegeneration) or the long-range association fibers such as the inferior/superior longitudinal fasciculi (vascular burden).

However, caution is needed when interpreting the longitudinal beneficial effects of physical activity on the risk of cognitive decline and dementia since several studies have found that when longer follow ups (over 10 years) are carried out, the protective effect of physical activity diminishes and becomes weak or non-significant (Blondell et al., 2014; Tan et al., 2016). The other DTI indices, instead, may provide additional and complementary information to support the interpretation of our findings. Moreover, the cross-sectional nature of this study prevents us from concluding whether physical activity-related changes were experience-dependent (ongoing changes) or were pre-existing structural changes that occurred over time. In fact, considering the lack of longitudinal data, it is not possible to ascertain whether these structural changes are due to high level of cardiorespiratory fitness that has either been maintained or achieved over the lifetime. Erickson et al. (2010) found that, comparing groups engaging in physical activity of variable intensity, the group who was highly physically active showed significant increases in gray matter volume compared with the other groups. The authors concluded that for any structural brain change to occur over time, the intensity of physical activity needs to be high. These findings are supported by a recent meta-analysis looking at the effects of physical exercise on cognitive function in ε4 carriers and non-carriers that found that, at low-to-moderate frequency/intensity interventions, cognitive performance was not much different between the two groups. However, at higher frequency/intensity interventions, the effect of physical activity on cognitive performance clearly seems to favor the non-carriers (Cancela-Carral et al., 2021).

### 4.1. Limitations

The first limitation of this study relates to the use of self-assessed measure of physical activity that may limit the investigation of the full impact of such lifestyle variable. It is also possible that a self-reported measure may not be sensitive enough to detect exercise with varying degree of duration, frequency and intensity that may affect cardiorespiratory fitness differentially. Moreover, defining physical activity as a binary variable hinders the possibility to detect dose-dependent effects on outcome measures of neurocognitive health (Tian et al., 2014).

In this study, an interaction effect between diagnostic group, physical activity and vascular burden was found for FA only. This may be due to the fact that FA, although sensitive to overall microstructural changes, is not specific and, hence, may not be very informative regarding the relative impact of the different factors investigated (Alexander et al., 2011). This is because recent studies have found that in cognitively impaired older adults, physical activity was directly associated with cerebral glucose metabolism irrespective of cardiovascular risk factors, whereas global gray matter volume was mediated by factors such as body mass index and insulin levels (Felisatti et al., 2022). The other problem that might have resulted in some bias in our study is that different participants have different levels of cardiorespiratory fitness and hence two people who might be classified as “highly active” might have different levels of cardiorespiratory fitness. Physical activity is also not consistent during longer periods for individuals who are classified as highly active during a particular period in time (Domingos et al., 2021). In other words, people do not always follow the same trajectory of exercise regimen during their course of time and this means that they might be highly active at one point and may not be as active in another period in time.

This lack of consistency could be one of the reasons why longitudinal studies examining physical activity seem to show inconclusive findings. Therefore, in their systematic review, Domingos et al. (2021) recommend that using objective measures (actigraphy) to monitor physical activity would be much more suitable to capture subtle patterns of everyday activities including intensity (low intensity activity) and duration that may not be captured from self-reported questionnaires that are limited in scope. The authors also suggest that a standardized criterion should be adopted across studies to measure physical activity. Nevertheless, the findings from cross-sectional studies in the extant literature show that physical activity in general does preserve WM microstructure and is beneficial for older adults who are at risk for dementia.

A further limitation, finally, is the unavailability of APOE-related data for a proportion of our cohort (see Table 1) that prevented us from including this variable as part of the inferential models.

### 4.2. Conclusion

The results suggested that physical activity was specifically beneficial for those participants at the highest risk such as those patients with high vascular burden, whereas this beneficial effect of physical activity was not detectable in controls.

While we used a validated standard questionnaire from the CAIDE to assess physical activity, it is a subjective tool and may not represent the best method to capture aspects such as duration, frequency and intensity. Future studies should address why these benefits are most notable among at risk groups for dementia as compared with healthy controls. Given that lifestyle interventions, such as physical activity, can result in WM structural changes across the lifespan, inducing WM plasticity changes as an alternative to pharmacological treatment becomes important during the preclinical stages. It is important to target at-risk individuals during the preclinical and subclinical phase when the disease has not completely manifested, thereby providing sufficient time to start an intervention program that would potentially delay the onset of AD and cognitive symptoms.

## Data availability statement

The raw data supporting the conclusions of this article will be made available by the authors, without undue reservation.

## Ethics statement

The studies involving human participants were reviewed and approved by the Regional Ethics Committee of Yorkshire and Humber (Ref No. 12/YH/0474) and the ethics committee of the Northern Savonia Hospital District. The patients/participants provided their written informed consent to participate in this study.

## Author contributions

SK conceived the study design, analyzed and interpreted the data, drafted, revised, and approved the final version of the manuscript for submission. RM, MDM, and HS contributed to data interpretation, revised, and approved the manuscript for submission. AV conceived the study, contributed to data collection, procurement of funding, data interpretation, revised, and finalized the manuscript for submission. All authors contributed to the article and approved the submitted version.

## Funding

This study was supported by the European Union Seventh Framework Programme (FP7/2007e2013) (grant agreement no. 601055, VPH-DARE@IT) to AV and HS.

## Conflict of interest

The authors declare that the research was conducted in the absence of any commercial or financial relationships that could be construed as a potential conflict of interest.

## Publisher’s note

All claims expressed in this article are solely those of the authors and do not necessarily represent those of their affiliated organizations, or those of the publisher, the editors and the reviewers. Any product that may be evaluated in this article, or claim that may be made by its manufacturer, is not guaranteed or endorsed by the publisher.
