# Supplementary material for: The effect of physical activity on white matter integrity in aging and prodromal to mild Alzheimer’s disease with vascular comorbidity
Source: Front Aging Neurosci. 2023 Jun 21;15:1096798. doi: 10.3389/fnagi.2023.1096798 (PMC10382177; doi:10.3389/fnagi.2023.1096798)
Supplement: Supplementary file 1 [file Data_Sheet_1.docx]

The Effect of Physical Activity on White Matter Integrity in Ageing and Prodromal to Mild Alzheimer’s Disease with Vascular Comorbidity

Supplementary Materials

**Srijan Konwar^1^, Riccardo Manca^1,2^, Matteo De Marco^1^, Hilkka Soininen^3^, Annalena Venneri^1,2^**

^1^Department of Life Sciences, Brunel University London, Uxbridge, UK

^2^Department of Medicine and Surgery, University of Parma, Parma, Italy

^3^Department of Neurology, University of Eastern Finland, Kuopio, Finland

*** Correspondence:**Annalena Venneri

a.venneri@sheffield.ac.uk

Table of Contents

[Supplementary Table S1. Comparison of Active and Inactive participants, irrespective of diagnostic status and vascular-burden scores 4](#_Toc133062113)

[Supplementary Table S2. Comparison of Controls and Patients, irrespective of active/inactive status and vascular-burden scores 5](#_Toc133062114)

[Supplementary Table S3. Comparison of participants with Low and High vascular-burden scores, irrespective of diagnostic and active/inactive status 6](#_Toc133062115)

[Supplementary Table S4. Significant differences in FA between diagnostic groups: Controls > Patients (FWE *p* < 0.05) 7](#_Toc133062116)

[Supplementary Table S5. Significant differences in AxD between diagnostic groups: Controls < Patients (FWE *p* < 0.05) 8](#_Toc133062117)

[Supplementary Table S6. Significant differences in MD between diagnostic groups: Controls < Patients (FWE *p* < 0.05) 10](#_Toc133062118)

[Supplementary Table S7. Significant differences in RD between diagnostic groups: Controls < Patients (FWE *p* < 0.05) 11](#_Toc133062119)

[Supplementary Table S8. Significant main effect of vascular burden on FA: Low Burden group > High Burden group (FWE *p* < 0.05) 12](#_Toc133062120)

[Supplementary Table S9. Significant main effect of Vascular Burden on AxD: High Burden group > Low Burden group (FWE *p* < 0.05) 13](#_Toc133062121)

[Supplementary Table S10. Significant main effect of Vascular Burden on MD: High Burden group > Low Burden group (FWE *p* < 0.05) 14](#_Toc133062122)

[Supplementary Table S11. Significant main effect of Vascular Burden on RD: High Burden group > Low Burden group (FWE *p* < 0.05) 15](#_Toc133062123)

[Supplementary Table S12. Significant main effect of Physical Activity on FA: Physically Active group > Non-physically Active group (FWE *p* < 0.05) 17](#_Toc133062124)

[Supplementary Table S13. Significant main effect of Physical Activity on AxD: Non-physically Active group > Physically Active group (FWE *p* < 0.05). 18](#_Toc133062125)

[Supplementary Table S14. Significant main effect of Physical Activity on MD: Non-physically Active group > Physically Active group (FWE *p* < 0.05). 19](#_Toc133062126)

[Supplementary Table S15. Significant main effect of Physical Activity on RD: Non-physically Active group > Physically Active group (FWE *p* < 0.05). 20](#_Toc133062127)

[Supplementary Table S16. Significant main effect of Physical Activity on FA after controlling for individual Vascular Risk Factors: Physically Active group > Non-physically Active group (FWE *p* < 0.05). 21](#_Toc133062128)

[Supplementary Table S17. Significant main effect of Physical Activity on AxD after controlling for individual Vascular Risk Factors: Non-physically Active group > Physically Active group (FWE *p* < 0.05) 22](#_Toc133062129)

[Supplementary Table S18. Significant main effect of Physical Activity on MD after controlling for individual Vascular Risk Factors: Non-physically Active group > Physically Active group (FWE *p* < 0.05) 23](#_Toc133062130)

[Supplementary Table S19. Significant main effect of Physical Activity on RD after controlling for individual Vascular Risk Factors: Non-physically Active group > Physically Active group (FWE *p* < 0.05) 24](#_Toc133062131)

[Supplementary Table S20. Interactive Effects of Diagnostic Group × Physical Activity × Vascular Burden show differences in FA in these WM tracts and regions after controlling for individual Vascular Risk Factors (FWE *p* < 0.05) 25](#_Toc133062132)

[Supplementary Table S21. *Post-hoc* analyses show that patients with High Vascular Burden who were active had higher FA than those who were inactive in these WM tracts after controlling for individual Vascular Risk Factors (FWE *p* < 0.0125) 27](#_Toc133062133)

[Supplementary Table S22. WM tracts with significantly higher FA values in physically active patients with high vascular burden compared with non-physically active controls with high vascular burden after controlling for individual Vascular Risk Factors (FWE *p* < 0.05) 28](#_Toc133062134)

Supplementary Table S1. Comparison of Active and Inactive participants, irrespective of diagnostic status and vascular-burden scores

| **Variables** | **Inactive**  **(*n* = 100)** | **Active (*n* = 113)** | **Test Statistics** | ***p*** |
| --- | --- | --- | --- | --- |
| *Demographic/Clinical* | | | | |
| **Age (years)^e,f^** | 72 (15) | 67 (16) | 4198.50 | **0.001** |
| **APOE status (Ɛ_4_ carriers %)^c,d^** | 31 (31%) | 46 (40.71%) | 0.959 | 0.327 |
| **BMI^e,f^** | 25.61 (5.61) | 26.53 (5.57) | 5360.50 | 0.519 |
| **Diabetes (Diabetic %)^c,d^** | 15 (15%) | 11 (9.73%) | 1.373 | 0.241 |
| **Education (years)^e,f^** | 11 (5) | 13 (6) | 4723.50 | **0.038** |
| **MMSE^e,f^** | 26 (5) | 28 (4) | 4582.50 | **0.017** |
| **Sex (Females %)^c,d^** | 62 (62%) | 55 (48.67%) | 3.806 | 0.051 |
| **Smoking (Smokers %)^c,d^** | 13 (13%) | 6 (5.31%) | 3.862 | **0.049** |
| **SBP^e,f^** | 140 (33) | 143 (22) | 5460.50 | 0.673 |
| **Vascular Scores^e,f^** | 30.34 (34.21) | 23.27 (18.46) | 4503 | **0.011** |
| *Neuroimaging* | | | | |
| **GMV (ml)^a,b^** | 573.11 (68.29) | 622.80 (72.30) | -5.137 | **< 0.001** |
| **WMV (ml)^a,b^** | 385.98 (47.46) | 420.38 (57.72) | -4.713 | **< 0.001** |
| **TIV (ml)^a,b^** | 1416.68 (147.30) | 1485.24 (153.95) | -3.310 | **0.001** |

To facilitate consultation, test statistics are reported only for significant between-group differences. APOE: Apolipoprotein E, BMI: Body Mass Index, GMV: Grey Matter Volume, MMSE: Mini Mental State Examination, SBP: Systolic Blood Pressure, TIV: Total Intracranial Volume, WMV: White Matter Volume; ^a^ Mean and standard deviation; ^b^ Independent Samples *t* test; ^c^ Frequencies; ^d^ Pearson’s *Chi-Square* test; ^e^ Median and interquartile range; ^f^ Mann–Whitney *U* test; All significance levels are at p < 0.05

Supplementary Table S2. Comparison of Controls and Patients, irrespective of active/inactive status and vascular-burden scores

| **Variables** | **Controls**  **(*n* = 77)** | **Patients (*n* = 136)** | **Test Statistics** | ***p*** |
| --- | --- | --- | --- | --- |
| *Demographic/Clinical* | | | | |
| **Age (years)^e,f^** | 69 (15) | 70 (17) | 4871 | 0.398 |
| **APOE status (Ɛ_4_ carriers %)^c,d^** | 14 (18.18%) | 63 (46.32%) | 15.452 | **< 0.001** |
| **BMI^e,f^** | 25.61 (5.61) | 26.53 (5.57) | 4542 | 0.108 |
| **Diabetes (Diabetic %)^c,d^** | 7 (9.09%) | 19 (13.97%) | 1.092 | 0.296 |
| **Education (years)^e,f^** | 14 (6) | 11 (6) | 3138 | **< 0.001** |
| **MMSE^e,f^** | 29 (2) | 25 (6) | 1766 | **< 0.001** |
| **Sex (Females %)^c,d^** | 49 (63.64%) | 68 (50%) | 3.693 | 0.055 |
| **Smoking (Smokers %)^c,d^** | 6 (7.79%) | 13 (9.56%) | 0.189 | 0.664 |
| **SBP^e,f^** | 143 (24) | 142 (29) | 5076 | 0.711 |
| **Vascular Scores^e,f^** | 26.12 (22.37) | 24.70 (28.02) | 4907 | 0.446 |
| *Neuroimaging* | | | | |
| **GMV (ml)^e,f^** | 613.86 (101.63) | 590.20 (104.35) | 3844 | **0.001** |
| **WMV (ml)^e,f^** | 409.55 (74.02) | 397.62 (79.96) | 4367 | **0.044** |
| **TIV (ml)^a,b^** | 1452.95 (164.54) | 1453.11 (148.93) | -0.008 | 0.994 |

To facilitate consultation, test statistics are reported only for significant between-group differences. APOE: Apolipoprotein E, BMI: Body Mass Index, GMV: Grey Matter Volume, MMSE: Mini Mental State Examination, SBP: Systolic Blood Pressure, TIV: Total Intracranial Volume, WMV: White Matter Volume; ^a^ Mean and standard deviation; ^b^ Independent-sample *t* test; ^c^ Frequencies; ^d^ Pearson’s *Chi-Square* test; ^e^ Median and interquartile range; ^f^ Mann–Whitney *U* test; all significance levels are at p < 0.05

**Supplementary Table S3.** Comparison of participants with Low and High vascular-burden scores, irrespective of diagnostic and active/inactive status

| **Variables** | **Low Burden**  **(*n* = 78)** | **High Burden (*n* = 135)** | **Test Statistics** | ***p*** |
| --- | --- | --- | --- | --- |
| *Demographic/Clinical* | | | | |
| **Age (years)^e,f^** | 60 (11) | 74 (12) | 1896.50 | **< 0.001** |
| **APOE status (Ɛ_4_ carriers %)^c,d^** | 32 (41.03%) | 45 (33.33%) | 0.255 | 0.614 |
| **BMI^e,f^** | 24.96 (7.70) | 26.50 (4.88) | 4946.50 | 0.462 |
| **Diabetes (Diabetic (%))^c,d^** | 0 (0%) | 26 (19.26%) | 17.111 | **< 0.001** |
| **Education (years)^e,f^** | 13 (5) | 11 (6) | 3361.50 | **< 0.001** |
| **MMSE^e,f^** | 27 (6) | 27 (4) | 4867.50 | 0.355 |
| **Sex (Females (%))^c,d^** | 54 (69.23%) | 63 (46.67%) | 10.167 | **0.001** |
| **Smoking (Smokers (%))^c,d^** | 5 (6.41%) | 14 (10.37%) | 0.954 | 0.329 |
| **SBP^e,f^** | 130.50 (22) | 149 (27) | 2674.50 | **< 0.001** |
| **Vascular Scores^e,f^** | 13.52 (6.90) | 35.45 (24.31) | 0.000 | **< 0.001** |
| *Neuroimaging* | | | | |
| **GMV (ml)^a,b^** | 627.93 (74.44) | 583.03 (69.75) | 4.416 | **< 0.001** |
| **WMV (ml)^a,b^** | 409.60 (54.74) | 401.12(56.30) | 1.069 | 0.286 |
| **TIV (ml)^e,f^** | 1463.44 (222.52) | 1433.54 (249.17) | 5136 | 0.766 |

To facilitate consultation, test statistics are reported only for significant between-group differences. APOE: Apolipoprotein E, BMI: Body Mass Index, GMV: Grey Matter Volume, MMSE: Mini Mental State Examination, SBP: Systolic Blood Pressure, TIV: Total Intracranial Volume, WMV: White Matter Volume; ^a^ Mean and standard deviation; ^b^ Independent Samples t test; ^c^ Frequencies; ^d^ Pearson’s *Chi-Square* test; ^e^ Median and interquartile range; ^f^ Mann–Whitney *U* test; All significance levels are at p < 0.05

Supplementary Table S4. Significant differences in FA between diagnostic groups: Controls > Patients (FWE *p* < 0.05)

|  |  |  |  | **MNI Coordinates of Local Maxima** | | | **White Matter Tracts** | | |
| --- | --- | --- | --- | --- | --- | --- | --- | --- | --- |
| **Clusters** | **Voxels** | **Value** | ***p*-value** | **X** | **Y** | **Z** | **JHU-ICBM-DTI-81 WM Labels** | **JHU-WM Tractography Atlas** | **Talairach Daemon** |
| 1 | 25579 | 5.15 | **0.005** | -41 | -55 | 38 | Unclassified |  | Left Cerebrum, Parietal Lobe, Angular Gyrus, White Matter |
|  |  | 5.08 | **0.005** | 14 | 35 | -10 | Unclassified | Right Uncinate Fasciculus |  |
|  |  | 4.85 | **0.005** | -47 | -59 | 5 | Unclassified |  | Left Cerebrum, Temporal Lobe, Middle Temporal Gyrus, White Matter |
|  |  | 4.83 | **0.005** | -40 | -53 | 36 | Unclassified | Left Superior Longitudinal Fasciculus |  |
|  |  | 4.82 | **0.005** | 0 | -20 | -8 | Unclassified |  | Left Brainstem, Midbrain |
|  |  | 4.78 | **0.005** | 20 | -87 | 11 | Unclassified | Forceps Major |  |
| 2 | 413 | 3.51 | **0.047** | -32 | 3 | 6 | Left External  Capsule | Left Superior Longitudinal Fasciculus |  |
|  |  | 3.33 | **0.047** | -31 | 2 | 8 | Left External Capsule | Left Superior Longitudinal Fasciculus |  |
|  |  | 3.32 | **0.047** | -31 | 5 | 7 | Left External Capsule |  |  |
|  |  | 3.19 | **0.047** | -31 | 7 | 5 | Left External Capsule |  |  |
|  |  | 3.07 | **0.047** | -32 | 5 | 3 | Left External Capsule | Left Superior Longitudinal Fasciculus |  |
|  |  | 2.84 | **0.047** | -27 | 18 | 2 | Left External Capsule | Left Inferior Fronto-Occipital Fasciculus |  |

Only tracts with > 100 voxels have been listed; Talairach Daemon Atlas was used when regions were either labelled as unclassified or undefined by JHU atlas, Covariates: Site of recruitment, total intracranial volume (TIV) and years of education

Supplementary Table S5. Significant differences in AxD between diagnostic groups: Controls < Patients (FWE *p* < 0.05)

|  |  |  |  | **MNI Coordinates of Local Maxima** | | | **White Matter Tracts** | | |
| --- | --- | --- | --- | --- | --- | --- | --- | --- | --- |
| **Clusters** | **Voxels** | **Value** | ***p*-value** | **X** | **Y** | **Z** | **JHU-ICBM-DTI-81 WM Labels** | **JHU-WM Tractography Atlas** | **Talairach Daemon** |
| 1 | 53308 | 6.55 | ***p* < 0.001** | 20 | -51 | 24 | Splenium of the Corpus Callosum |  |  |
|  |  | 5.93 | ***p* < 0.001** | 19 | -52 | 27 | Unclassified |  | Right Cerebrum, Parietal Lobe, Sub-Gyral, White Matter |
|  |  | 5.74 | ***p* < 0.001** | -12 | -28 | 13 | Unclassified | Left Anterior Thalamic Radiation |  |
|  |  | 5.68 | ***p* < 0.001** | -35 | -36 | 24 | Unclassified | Left Superior Longitudinal Fasciculus |  |
|  |  | 5.67 | ***p* < 0.001** | -13 | -30 | 13 | Unclassified | Left Anterior Thalamic Radiation |  |
|  |  | 5.53 | ***p* < 0.001** | -21 | -36 | 7 | Unclassified | Left Anterior Thalamic Radiation |  |
| 2 | 185 | 3.86 | **0.048** | 11 | -76 | 21 | Unclassified |  | Right Cerebrum, Occipital Lobe, Cuneus, White Matter |
|  |  | 2.51 | **0.048** | 13 | -77 | 23 | Unclassified |  | Right Cerebrum, Occipital Lobe, Cuneus, White Matter |
|  |  | 2.34 | **0.048** | 16 | -77 | 18 | Unclassified | Forceps Major |  |
|  |  | 2.27 | **0.048** | 17 | -79 | 19 | Unclassified | Forceps Major |  |
|  |  | 2.26 | **0.048** | 21 | -81 | 24 | Unclassified | Forceps Major |  |
|  |  | 2.17 | **0.048** | 10 | -79 | 24 | Unclassified |  | Right Cerebrum, Occipital Lobe, Cuneus, White Matter |
| 3 | 148 | 3 | **0.05** | -34 | -70 | 0 | Unclassified | Left Inferior Longitudinal Fasciculus |  |
|  |  | 2.59 | **0.05** | -30 | -67 | -10 | Unclassified | Left Inferior Longitudinal Fasciculus |  |
|  |  | 2.25 | **0.05** | -29 | -71 | -10 | Unclassified | Left Inferior Longitudinal Fasciculus |  |
|  |  | 2.08 | **0.05** | -34 | -75 | -6 | Unclassified | Left Inferior Longitudinal Fasciculus |  |
|  |  | 2.01 | **0.05** | -33 | -72 | -9 | Unclassified | Left Inferior Longitudinal Fasciculus |  |
|  |  | 1.94 | **0.05** | -35 | -73 | -8 | Unclassified |  | Left Cerebrum, Occipital Lobe, Sub-Gyral, White Matter |

Only tracts with > 100 voxels have been listed; Talairach Daemon Atlas was used when regions were either labelled as unclassified or undefined by JHU Atlas, Covariates: Site of recruitment, total intracranial volume (TIV) and years of education

Supplementary Table S6. Significant differences in MD between diagnostic groups: Controls < Patients (FWE *p* < 0.05)

|  |  |  |  | **MNI Coordinates of Local Maxima** | | | **White Matter Tracts** | | |
| --- | --- | --- | --- | --- | --- | --- | --- | --- | --- |
| **Clusters** | **Voxels** | **Value** | ***p*-value** | **X** | **Y** | **Z** | **JHU-ICBM-DTI-81 WM Labels** | **JHU-WM Tractography Atlas** | **Talairach Daemon** |
| 1 | 68088 | 6.42 | ***p* < 0.001** | 19 | -51 | 25 | Splenium of the Corpus Callosum |  |  |
|  |  | 6.36 | ***p* < 0.001** | 19 | -52 | 27 | Unclassified |  | Right Cerebrum, Parietal Lobe, Sub-Gyral, White Matter |
|  |  | 5.89 | ***p* < 0.001** | 23 | -51 | 26 | Right Posterior Corona Radiata | Right Inferior Fronto-Occipital Fasciculus/Right Anterior Thalamic Radiation |  |
|  |  | 5.88 | ***p* < 0.001** | 11 | -37 | 8 | Splenium of the Corpus Callosum |  |  |
|  |  | 5.82 | ***p* < 0.001** | -13 | -30 | 13 | Unclassified | Left Anterior Thalamic Radiation |  |
|  |  | 5.71 | ***p* < 0.001** | 23 | -48 | 26 | Unclassified |  | Right Cerebrum, Sub-Lobar, Extra-Nuclear, White Matter |

Talairach Daemon Atlas was used when regions were either labelled as unclassified or undefined by JHU Atlas, Covariates: Site of recruitment, total intracranial volume (TIV) and years of education

Supplementary Table S7. Significant differences in RD between diagnostic groups: Controls < Patients (FWE *p* < 0.05)

|  |  |  |  | **MNI Coordinates of Local Maxima** | | | **White Matter Tracts** | | |
| --- | --- | --- | --- | --- | --- | --- | --- | --- | --- |
| **Clusters** | **Voxels** | **Value** | ***p*-value** | **X** | **Y** | **Z** | **JHU-ICBM-DTI-81 WM Labels** | **JHU-WM Tractography Atlas** | **Talairach Daemon** |
| 1 | 63209 | 5.8 | ***p* < 0.001** | -12 | -29 | 13 | Unclassified | Left Anterior Thalamic Radiation |  |
|  |  | 5.72 | ***p* < 0.001** | 20 | -52 | 26 | Unclassified |  | Right Cerebrum, Parietal Lobe, Sub-Gyral, White Matter |
|  |  | 5.68 | ***p* < 0.001** | 11 | -37 | 8 | Splenium of the Corpus Callosum |  |  |
|  |  | 5.53 | ***p* < 0.001** | -21 | -36 | 7 | Unclassified | Left Anterior Thalamic Radiation |  |
|  |  | 5.4 | ***p* < 0.001** | -8 | -22 | 14 | Unclassified | Left Anterior Thalamic Radiation |  |
|  |  | 5.38 | ***p* < 0.001** | -4 | -11 | 13 | Unclassified | Left Anterior Thalamic Radiation |  |
| 2 | 326 | 4.24 | **0.045** | 8 | -3 | 35 | Right Cingulum (Cingulate Gyrus) | Right Cingulum (Cingulate Gyrus) |  |
|  |  | 3.97 | **0.045** | 10 | 21 | 24 | Right Cingulum (Cingulate Gyrus) | Right Cingulum (Cingulate Gyrus) |  |
|  |  | 3.89 | **0.045** | 8 | -5 | 37 | Right Cingulum (Cingulate Gyrus) | Right Cingulum (Cingulate Gyrus) |  |
|  |  | 3.85 | **0.045** | 7 | -1 | 35 | Right Cingulum (Cingulate Gyrus) | Right Cingulum (Cingulate Gyrus) |  |
|  |  | 3.8 | **0.045** | 10 | 18 | 27 | Right Cingulum (Cingulate Gyrus) | Right Cingulum (Cingulate Gyrus) |  |
|  |  | 3.74 | **0.045** | 9 | -12 | 35 | Right Cingulum (Cingulate Gyrus) | Right Cingulum (Cingulate Gyrus) |  |

Only tracts with > 100 voxels have been listed; Talairach Daemon Atlas was used when regions were either labelled as unclassified or undefined by JHU Atlas, Covariates: Site of recruitment, total intracranial volume (TIV) and years of education

Supplementary Table S8. Significant main effect of vascular burden on FA: Low Burden group > High Burden group (FWE *p* < 0.05)

|  |  |  |  | **MNI Coordinates of Local Maxima** | | | **White Matter Tracts** | |
| --- | --- | --- | --- | --- | --- | --- | --- | --- |
| **Clusters** | **Voxels** | **Value** | ***p*-value** | **X** | **Y** | **Z** | **JHU-ICBM-DTI-81 WM Labels** | **JHU-WM Tractography Atlas** |
| 1 | 30559 | 4.98 | **0.002** | -15 | 37 | 1 | Left Anterior Corona Radiata | Forceps Minor |
|  |  | 4.93 | **0.002** | -15 | 35 | -1 | Left Anterior Corona Radiata | Forceps Minor |
|  |  | 4.88 | **0.002** | -20 | -21 | 39 | Left Superior Corona Radiata | Left Corticospinal Tract |
|  |  | 4.83 | **0.002** | -24 | -35 | 46 | Unclassified | Left Corticospinal Tract |
|  |  | 4.79 | **0.002** | -19 | 34 | 13 | Left Anterior Corona Radiata | Forceps Minor |
|  |  | 4.78 | **0.002** | -15 | 37 | -3 | Left Anterior Corona Radiata | Forceps Minor |

Covariates: Site of recruitment, total intracranial volume (TIV) and years of education

Supplementary Table S9. Significant main effect of Vascular Burden on AxD: High Burden group > Low Burden group (FWE *p* < 0.05)

|  |  |  |  | **MNI Coordinates of Local Maxima** | | | **White Matter Tracts** | | |
| --- | --- | --- | --- | --- | --- | --- | --- | --- | --- |
| **Clusters** | **Voxels** | **Value** | ***p*-value** | **X** | **Y** | **Z** | **JHU-ICBM-DTI-81 WM Labels** | **JHU-WM Tractography Atlas** | **Talairach Daemon** |
| 1 | 34302 | 5.81 | **0.001** | -24 | -5 | 19 | Left Superior Corona Radiata |  |  |
|  |  | 5.35 | **0.001** | -27 | 12 | 15 | Unclassified |  | Left Cerebrum, Sub-Lobar, Extra Nuclear, White Matter |
|  |  | 5.14 | **0.001** | -22 | -2 | 16 | Left Anterior Limb of Internal Capsule |  |  |
|  |  | 5 | **0.001** | -27 | 13 | 13 | Unclassified |  | Left Cerebrum, Sub-Lobar, Extra Nuclear, White Matter |
|  |  | 4.97 | **0.001** | -22 | 16 | 14 | Left Anterior Corona Radiata | Left Anterior Thalamic Radiation |  |
|  |  | 4.96 | **0.001** | -26 | -17 | 24 | Left Superior Corona Radiata | Left Corticospinal Tract |  |

Talairach Daemon Atlas was used when regions were either labelled as unclassified or undefined by JHU Atlas, Covariates: Site of recruitment, total intracranial volume (TIV) and years of education

Supplementary Table S10. Significant main effect of Vascular Burden on MD: High Burden group > Low Burden group (FWE *p* < 0.05)

|  |  |  |  | **MNI Coordinates of Local Maxima** | | | **White Matter Tracts** | |
| --- | --- | --- | --- | --- | --- | --- | --- | --- |
| **Clusters** | **Voxels** | **Value** | ***p*-value** | **X** | **Y** | **Z** | **JHU-ICBM-DTI-81 WM Labels** | **JHU-WM Tractography Atlas** |
| 1 | 51651 | 5.51 | **0.001** | -22 | -3 | 15 | Left Anterior Limb of Internal Capsule | Left Anterior Thalamic Radiation |
|  |  | 5.47 | **0.001** | -22 | -4 | 17 | Left Anterior Limb of Internal Capsule | Left Anterior Thalamic  Radiation |
|  |  | 5.28 | **0.001** | -26 | -11 | 18 | Left Posterior Limb of Internal Capsule | Left Corticospinal tract |
|  |  | 5.28 | **0.001** | -23 | -4 | 19 | Left Superior Corona Radiata |  |
|  |  | 5.25 | **0.001** | -27 | -24 | 27 | Left Posterior Corona Radiata | Left Corticospinal tract |
|  |  | 5.23 | **0.001** | -24 | -9 | 17 | Left Posterior Limb of Internal Capsule |  |

Covariates: Site of recruitment, total intracranial volume (TIV) and years of education

Supplementary Table S11. Significant main effect of Vascular Burden on RD: High Burden group > Low Burden group (FWE *p* < 0.05)

|  |  |  |  | **MNI Coordinates of Local Maxima** | | | **White Matter Tracts** | | |
| --- | --- | --- | --- | --- | --- | --- | --- | --- | --- |
| **Clusters** | **Voxels** | **Value** | ***p*-value** | **X** | **Y** | **Z** | **JHU-ICBM-DTI-81 WM Labels** | **JHU-WM Tractography Atlas** | **Talairach Daemon** |
| 1 | 50343 | 4.96 | **0.001** | -31 | -8 | 12 | Left External Capsule | Left Superior Longitudinal Fasciculus |  |
|  |  | 4.72 | **0.001** | 36 | -57 | 7 | Right Posterior Thalamic Radiation (include Optic Radiation) | Right Inferior Longitudinal Fasciculus |  |
|  |  | 4.68 | **0.001** | -16 | -4 | 50 | Unclassified |  | Left Cerebrum, Frontal Lobe, Medial Frontal Gyrus, White Matter |
|  |  | 4.65 | **0.001** | 20 | 35 | 15 | Right Anterior Corona Radiata | Forceps Minor |  |
|  |  | 4.63 | **0.001** | -25 | 24 | 16 | Left Anterior Corona Radiata | Left Inferior Fronto-Occipital Fasciculus |  |
|  |  | 4.6 | **0.001** | 0 | 17 | -2 | Unclassified |  | Left Cerebrum, Sub-Lobar, Extra-Nuclear, White Matter |
| 2 | 105 | 2.76 | **0.05** | 51 | -18 | -17 | Unclassified | Right Inferior Longitudinal Fasciculus |  |
|  |  | 2.51 | **0.05** | 49 | -21 | -19 | Unclassified | Right Inferior Longitudinal Fasciculus |  |
|  |  | 2.47 | **0.05** | 54 | -14 | -19 | Unclassified |  | Right Cerebrum, Temporal Lobe, Sub-Gyral, White Matter |
|  |  | 2.47 | **0.05** | 52 | -15 | -19 | Unclassified |  | Right Cerebrum, Temporal Lobe, Sub-Gyral, White Matter |
|  |  | 2.42 | **0.05** | 57 | -18 | -16 | Unclassified |  | Right Cerebrum, Temporal Lobe, Middle Temporal Gyrus, White Matter |
|  |  | 2.33 | **0.05** | 54 | -18 | -17 | Unclassified |  | Right Cerebrum, Temporal Lobe, Middle Temporal Gyrus, White Matter |

Only tracts with > 100 voxels have been listed; Talairach Daemon Atlas was used when regions were either labelled as unclassified or undefined by JHU Atlas, Covariates: Site of recruitment, total intracranial volume (TIV) and years of education

Supplementary Table S12. Significant main effect of Physical Activity on FA: Physically Active group > Non-physically Active group (FWE *p* < 0.05)

|  |  |  |  | **MNI Coordinates of Local Maxima** | | | **White Matter Tracts** | |
| --- | --- | --- | --- | --- | --- | --- | --- | --- |
| **Clusters** | **Voxels** | **Value** | ***p*-value** | **X** | **Y** | **Z** | **JHU-ICBM-DTI-81 WM Labels** | **JHU-WM Tractography Atlas** |
| 1 | 74220 | 5.67 | **0.001** | 28 | -26 | -7 | Right Fornix (Cres)/Stria Terminalis (cannot be resolved with current resolution) |  |
|  |  | 5.45 | **0.001** | 11 | -41 | 31 | Unclassified | Right Cingulum (Cingulate Gyrus) |
|  |  | 5.41 | **0.001** | -11 | -42 | 31 | Unclassified | Left Cingulum (Cingulate Gyrus) |
|  |  | 5.15 | **0.001** | 50 | -48 | -10 | Unclassified | Right Superior Longitudinal Fasciculus (Temporal Part) |
|  |  | 5.07 | **0.001** | 10 | -46 | 27 | Right Cingulum (Cingulate Gyrus) | Right Cingulum (Cingulate Gyrus) |
|  |  | 5.04 | **0.001** | 10 | -37 | 33 | Right Cingulum (Cingulate Gyrus) | Right Cingulum (Cingulate Gyrus) |

Covariates: Site of recruitment, total intracranial volume (TIV) and years of education

Supplementary Table S13. Significant main effect of Physical Activity on AxD: Non-physically Active group > Physically Active group (FWE *p* < 0.05).

|  |  |  |  | **MNI Coordinates of Local Maxima** | | | **White Matter Tracts** | |
| --- | --- | --- | --- | --- | --- | --- | --- | --- |
| **Clusters** | **Voxels** | **Value** | ***p*-value** | **X** | **Y** | **Z** | **JHU-ICBM-DTI-81 WM Labels** | **JHU-WM Tractography Atlas** |
| 1 | 25047 | 4.45 | **0.01** | 30 | -32 | 11 | Right Retrolenticular Part of Internal Capsule | Right Inferior Fronto-Occipital Fasciculus |
|  |  | 4.28 | **0.01** | -37 | -50 | -2 | Left Posterior Thalamic Radiation (include Optic Radiation) | Left Inferior Fronto-Occipital Fasciculus |
|  |  | 4.27 | **0.01** | -37 | -1 | -28 | Unclassified | Left Inferior Longitudinal Fasciculus |
|  |  | 4.24 | **0.01** | -39 | -49 | -3 | Left Posterior Thalamic Radiation (include Optic Radiation) | Left Inferior Longitudinal Fasciculus |
|  |  | 4.2 | **0.01** | -39 | -47 | -5 | Left Sagittal Stratum (include Inferior Longitudinal Fasciculus and Inferior Fronto-Occipital Fasciculus) | Left Inferior Longitudinal Fasciculus |
|  |  | 4.12 | **0.01** | -37 | -2 | -26 | Unclassified | Left Uncinate Fasciculus |

Covariates: Site of recruitment, total intracranial volume (TIV) and years of education

Supplementary Table S14. Significant main effect of Physical Activity on MD: Non-physically Active group > Physically Active group (FWE *p* < 0.05).

|  |  |  |  | **MNI Coordinates of Local Maxima** | | | **White Matter Tracts** | | |
| --- | --- | --- | --- | --- | --- | --- | --- | --- | --- |
| **Clusters** | **Voxels** | **Value** | ***p*-value** | **X** | **Y** | **Z** | **JHU-ICBM-DTI-81 WM Labels** | **JHU-WM Tractography Atlas** | **Talairach Daemon** |
| 1 | 63002 | 5.9 | **0.001** | -11 | -44 | 27 | Left Cingulum (Cingulate Gyrus) | Left Cingulum (Cingulate Gyrus) |  |
|  |  | 5.47 | **0.001** | -38 | 0 | -29 | Unclassified | Left Inferior Longitudinal Fasciculus |  |
|  |  | 5.15 | **0.001** | 39 | -53 | 14 | Right Superior Longitudinal Fasciculus | Right Superior Longitudinal Fasciculus |  |
|  |  | 4.94 | **0.001** | 31 | -34 | 15 | Right Retrolenticular part of Internal Capsule |  |  |
|  |  | 4.9 | **0.001** | -14 | -61 | 35 | Unclassified |  | Left Cerebrum, Parietal Lobe, Precuneus, White Matter |
|  |  | 4.85 | **0.001** | -31 | -35 | 12 | Left Retrolenticular part of Internal Capsule |  |  |

Talairach Daemon Atlas was used when regions were either labelled as unclassified or undefined by JHU Atlas, Covariates: Site of recruitment, total intracranial volume (TIV) and years of education

Supplementary Table S15. Significant main effect of Physical Activity on RD: Non-physically Active group > Physically Active group (FWE *p* < 0.05).

|  |  |  |  | **MNI Coordinates of Local Maxima** | | | **White Matter Tracts** | |
| --- | --- | --- | --- | --- | --- | --- | --- | --- |
| **Clusters** | **Voxels** | **Value** | ***p*-value** | **X** | **Y** | **Z** | **JHU-ICBM-DTI-81 WM Labels** | **JHU-WM Tractography Atlas** |
| 1 | 72872 | 5.77 | ***p* < 0.001** | -12 | -43 | 28 | Unclassified | Left Cingulum (Cingulate Gyrus) |
|  |  | 5.63 | ***p* < 0.001** | -38 | 0 | -29 | Unclassified | Left Inferior Longitudinal Fasciculus |
|  |  | 5.6 | ***p* < 0.001** | -13 | -62 | 34 | Unclassified | Left Cingulum (Cingulate Gyrus) |
|  |  | 5.37 | ***p* < 0.001** | -11 | -46 | 29 | Unclassified | Left Cingulum (Cingulate Gyrus) |
|  |  | 5.15 | ***p* < 0.001** | -41 | -1 | -30 | Unclassified | Left Inferior Longitudinal Fasciculus |
|  |  | 4.99 | ***p* < 0.001** | -44 | 4 | 16 | Unclassified | Left Superior Longitudinal Fasciculus |

Covariates: Site of recruitment, total intracranial volume (TIV) and years of education

Supplementary Table S16. Significant main effect of Physical Activity on FA after controlling for individual Vascular Risk Factors: Physically Active group > Non-physically Active group (FWE *p* < 0.05).

|  |  |  |  | **MNI Coordinates of Local Maxima** | | | **White Matter Tracts** | | |
| --- | --- | --- | --- | --- | --- | --- | --- | --- | --- |
| **Clusters** | **Voxels** | **Value** | ***p*-value** | **X** | **Y** | **Z** | **JHU-ICBM-DTI-81 WM Labels** | **JHU-WM Tractography Atlas** | **Talairach Daemon** |
| 1 | 76955 | 5.83 | **0.001** | 11 | -41 | 31 | Unclassified | Right Cingulum (Cingulate Gyrus) |  |
|  | 76955 | 5.6 | **0.001** | 28 | -26 | -7 | Right Fornix (Cres)/Stria Terminalis (cannot be resolved with current resolution) |  |  |
|  | 76955 | 5.33 | **0.001** | -11 | -42 | 31 | Unclassified | Left Cingulum (Cingulate Gyrus) |  |
|  | 76955 | 5.27 | **0.001** | 30 | -23 | -7 | Right Fornix (Cres)/Stria Terminalis (cannot be resolved with current resolution) |  |  |
|  | 76955 | 5.23 | **0.001** | -19 | -57 | 36 | Unclassified |  | Left Cerebrum, Parietal Lobe, Precuneus, White Matter |
|  | 76955 | 5.18 | **0.001** | -34 | -25 | -1 | Left Retrolenticular Part of Internal Capsule | Left Inferior Fronto-Occipital Fasciculus |  |

Talairach Daemon Atlas was used when regions were either labelled as unclassified or undefined by JHU atlas, Covariates: Age, body-mass index, diabetes, site of recruitment, smoking status, systolic blood pressure, total intracranial volume (TIV) and years of education

Supplementary Table S17. Significant main effect of Physical Activity on AxD after controlling for individual Vascular Risk Factors: Non-physically Active group > Physically Active group (FWE *p* < 0.05)

|  |  |  |  | **MNI Coordinates of Local Maxima** | | | **White Matter Tracts** | | |
| --- | --- | --- | --- | --- | --- | --- | --- | --- | --- |
| **Clusters** | **Voxels** | **Value** | ***p*-value** | **X** | **Y** | **Z** | **JHU-ICBM-DTI-81 WM Labels** | **JHU-WM Tractography Atlas** | **Talairach Daemon** |
| 1 | 24860 | 4.51 | **0.008** | 30 | -32 | 11 | Right Retrolenticular Part of Internal Capsule | Right Inferior Fronto-Occipital Fasciculus |  |
|  | 24860 | 4.32 | **0.008** | -37 | -50 | -2 | Left Posterior Thalamic Radiation (include Optic Radiation) | Left Inferior Fronto-Occipital Fasciculus |  |
|  | 24860 | 4.29 | **0.008** | -39 | -49 | -3 | Left Posterior Thalamic Radiation (include Optic Radiation) | Left Inferior Longitudinal Fasciculus |  |
|  | 24860 | 4.27 | **0.008** | -39 | -47 | -5 | Left Sagittal Stratum (include Inferior Longitudinal Fasciculus and Inferior Fronto-Occipital Fasciculus | Left Inferior Longitudinal Fasciculus |  |
|  | 24860 | 4.18 | **0.008** | -38 | 0 | -29 | Unclassified | Left Inferior Longitudinal Fasciculus |  |
|  | 24860 | 4.15 | **0.008** | 21 | -33 | 0 | Unclassified |  | Right Cerebrum, Sub-Lobar, Extra-Nuclear, White Matter |

Talairach Daemon Atlas was used when regions were either labelled as unclassified or undefined by JHU atlas, Covariates: Age, body-mass index, diabetes, site of recruitment, smoking status, systolic blood pressure, total intracranial volume (TIV) and years of education

Supplementary Table S18. Significant main effect of Physical Activity on MD after controlling for individual Vascular Risk Factors: Non-physically Active group > Physically Active group (FWE *p* < 0.05)

|  |  |  |  | **MNI Coordinates of Local Maxima** | | | **White Matter Tracts** | | |
| --- | --- | --- | --- | --- | --- | --- | --- | --- | --- |
| **Clusters** | **Voxels** | **Value** | ***p*-value** | **X** | **Y** | **Z** | **JHU-ICBM-DTI-81 WM Labels** | **JHU-WM Tractography Atlas** | **Talairach Daemon** |
| 1 | 67511 | 5.76 | ***p* < 0.001** | -38 | 0 | -29 | Unclassified | Left Inferior Longitudinal Fasciculus |  |
|  | 67511 | 5.67 | ***p* < 0.001** | -11 | -44 | 27 | Left Cingulum (Cingulate Gyrus) | Left Cingulum (Cingulate Gyrus) |  |
|  | 67511 | 5.19 | ***p* < 0.001** | 39 | -53 | 14 | Right Superior Longitudinal Fasciculus | Right Superior Longitudinal Fasciculus |  |
|  | 67511 | 5.06 | ***p* < 0.001** | 31 | -34 | 15 | Right Retrolenticular Part of Internal Capsule |  |  |
|  | 67511 | 4.98 | ***p* < 0.001** | -14 | -61 | 34 | Unclassified |  | Left Cerebrum, Parietal Lobe, Precuneus, White Matter |
|  | 67511 | 4.94 | ***p* < 0.001** | 37 | -37 | 7 | Right Retrolenticular Part of Internal Capsule | Right Inferior Longitudinal Fasciculus/Right Inferior Fronto-Occipital Fasciculus |  |

Talairach Daemon Atlas was used when regions were either labelled as unclassified or undefined by JHU atlas, Covariates: Age, body-mass index, diabetes, site of recruitment, smoking status, systolic blood pressure, total intracranial volume (TIV) and years of education

Supplementary Table S19. Significant main effect of Physical Activity on RD after controlling for individual Vascular Risk Factors: Non-physically Active group > Physically Active group (FWE *p* < 0.05)

|  |  |  |  | **MNI Coordinates of Local Maxima** | | | **White Matter Tracts** | |
| --- | --- | --- | --- | --- | --- | --- | --- | --- |
| **Clusters** | **Voxels** | **Value** | ***p*-value** | **X** | **Y** | **Z** | **JHU-ICBM-DTI-81 WM Labels** | **JHU-WM Tractography Atlas** |
| 1 | 75299 | 5.9 | ***p* < 0.001** | -38 | 0 | -29 | Unclassified | Left Inferior Longitudinal Fasciculus |
|  | 75299 | 5.76 | ***p* < 0.001** | -12 | -43 | 28 | Unclassified | Left Cingulum (Cingulate Gyrus) |
|  | 75299 | 5.48 | ***p* < 0.001** | -13 | -62 | 34 | Unclassified | Left Cingulum (Cingulate Gyrus) |
|  | 75299 | 5.19 | ***p* < 0.001** | -41 | -1 | -30 | Unclassified | Left Inferior Longitudinal Fasciculus |
|  | 75299 | 5.14 | ***p* < 0.001** | -10 | -46 | 28 | Unclassified | Left Cingulum (Cingulate Gyrus) |
|  | 75299 | 5.1 | ***p* < 0.001** | 39 | -53 | 14 | Right Superior Longitudinal Fasciculus | Right Superior Longitudinal Fasciculus |

Covariates: Age, body-mass index, diabetes, site of recruitment, smoking status, systolic blood pressure, total intracranial volume (TIV) and years of education

Supplementary Table S20. Interactive Effects of Diagnostic Group × Physical Activity × Vascular Burden show differences in FA in these WM tracts and regions after controlling for individual Vascular Risk Factors (FWE *p* < 0.05)

|  |  |  |  | **MNI Coordinates of Local Maxima** | | | **WM Tracts** | |  |
| --- | --- | --- | --- | --- | --- | --- | --- | --- | --- |
| **Clusters** | **Voxels** | **Value** | ***p*-value** | **X** | **Y** | **Z** | **JHU-ICBM-DTI-81 WM Labels** | **JHU-WM Tractography Atlas** | **Talairach Daemon** |
| 5 | 1501 | 4.32 | **0.039** | 27 | 38 | 8 | Unclassified | Right Anterior Thalamic Radiation |  |
|  | 1501 | 3.83 | **0.039** | 18 | 30 | 23 | Right Anterior Corona Radiata | Forceps Minor |  |
|  | 1501 | 3.68 | **0.039** | 18 | 24 | 32 | Unclassified |  | Right Cerebrum, Frontal Lobe, Cingulate Gyrus, White Matter |
|  | 1501 | 3.68 | **0.039** | 21 | 31 | 21 | Right Anterior Corona Radiata | Forceps Minor |  |
|  | 1501 | 3.65 | **0.039** | 32 | 40 | 8 | Unclassified | Right Anterior Thalamic Radiation |  |
|  | 1501 | 3.63 | **0.039** | 24 | 35 | 4 | Right Anterior Corona Radiata | Right Inferior Fronto-Occipital Fasciculus |  |
| 4 | 282 | 3.7 | **0.047** | 28 | -8 | 24 | Right Superior Corona Radiata | Right Corticospinal Tract |  |
|  | 282 | 3.65 | **0.047** | 28 | -6 | 23 | Right Superior Corona Radiata | Right Superior Longitudinal Fasciculus |  |
|  | 282 | 2.77 | **0.047** | 26 | -6 | 25 | Right Superior Corona Radiata | Right Corticospinal Tract |  |
|  | 282 | 2.67 | **0.047** | 25 | -8 | 34 | Right Superior Corona Radiata |  |  |
|  | 282 | 2.63 | **0.047** | 28 | -2 | 23 | Right Superior Corona Radiata | Right Superior Longitudinal Fasciculus |  |
|  | 282 | 2.53 | **0.047** | 25 | -8 | 25 | Right Superior Corona Radiata |  |  |
| 3 | 153 | 3.11 | **0.049** | 14 | 40 | -14 | Unclassified | Right Uncinate Fasciculus |  |
|  | 153 | 2.92 | **0.049** | 16 | 39 | -8 | Right Anterior Corona Radiata | Right Inferior Fronto-Occipital Fasciculus |  |
|  | 153 | 2.64 | **0.049** | 19 | 42 | -4 | Unclassified | Right Anterior Thalamic Radiation |  |
|  | 153 | 2.61 | **0.049** | 22 | 33 | -7 | Right Anterior Corona Radiata | Right Inferior Fronto-Occipital Fasciculus |  |
|  | 153 | 2.56 | **0.049** | 14 | 34 | -12 | Unclassified | Right Uncinate Fasciculus |  |
|  | 153 | 2.51 | **0.049** | 18 | 40 | -5 | Unclassified | Right Uncinate Fasciculus |  |
| 2 | 113 | 3.62 | **0.048** | 22 | -48 | 40 | Unclassified |  | Right Cerebrum, Parietal Lobe, Precuneus, White Matter |
|  | 113 | 3.23 | **0.048** | 20 | -50 | 41 | Unclassified |  | Right Cerebrum, Parietal Lobe, Precuneus, White Matter |
|  | 113 | 3.23 | **0.048** | 21 | -57 | 35 | Unclassified | Right Inferior Fronto-Occipital Fasciculus |  |
|  | 113 | 2.93 | **0.048** | 21 | -58 | 33 | Unclassified | Right Inferior Longitudinal Fasciculus/Right Inferior Fronto-Occipital Fasciculus |  |
|  | 113 | 2.81 | **0.048** | 26 | -49 | 37 | Unclassified |  | Right Cerebrum, Parietal Lobe, Sub-Gyral, White Matter |
|  | 113 | 2.75 | **0.048** | 19 | -56 | 38 | Unclassified |  | Right Cerebrum, Parietal Lobe, Precuneus, White Matter |

Only tracts with > 100 voxels have been listed; Talairach Daemon Atlas was used when regions were either labelled as unclassified or undefined by JHU Atlas, Covariates: Age, body-mass index, diabetes, site of recruitment, smoking status, systolic blood pressure, total intracranial volume (TIV) and years of education

Supplementary Table S21. *Post-hoc* analyses show that patients with High Vascular Burden who were active had higher FA than those who were inactive in these WM tracts after controlling for individual Vascular Risk Factors (FWE *p* < 0.0125)

|  |  |  |  | **MNI Coordinates of Local Maxima** | | | **WM Tracts** | |  |
| --- | --- | --- | --- | --- | --- | --- | --- | --- | --- |
| **Clusters** | **Voxels** | **Value** | ***p*-value** | **X** | **Y** | **Z** | **JHU-ICBM-DTI-81 WM Labels** | **JHU-WM Tractography Atlas** | **Talairach Daemon** |
| 1 | 69952 | 6.26 | ***p* < 0.001** | 28 | -26 | -7 | Right Fornix (Cres)/Stria Terminalis (cannot be resolved with current resolution) |  |  |
|  | 69952 | 6.03 | ***p* < 0.001** | 32 | -58 | 11 | Right Posterior Thalamic Radiation (include Optic Radiation) | Forceps Major |  |
|  | 69952 | 5.86 | ***p* < 0.001** | 33 | -57 | 15 | Right Posterior Thalamic Radiation (include Optic Radiation) | Right Inferior Fronto-Occipital Fasciculus |  |
|  | 69952 | 5.85 | ***p* < 0.001** | 33 | -67 | 18 | Unclassified | Right Inferior Fronto-Occipital Fasciculus |  |
|  | 69952 | 5.79 | ***p* < 0.001** | 16 | -3 | 52 | Unclassified |  | Right Cerebrum, Frontal Lobe, Medial Frontal Gyrus, White Matter |
|  | 69952 | 5.76 | ***p* < 0.001** | 28 | -8 | 23 | Right Superior Corona Radiata | Right Superior Longitudinal Fasciculus |  |

Bonferroni correction for multiple comparisons was applied (corrected *p* < 0.0125); Talairach Daemon Atlas was used when regions were either labelled as unclassified or undefined by JHU Atlas, Covariates: Age, body-mass index, diabetes, site of recruitment, smoking status, systolic blood pressure, total intracranial volume (TIV) and years of education

Supplementary Table S22. WM tracts with significantly higher FA values in physically active patients with high vascular burden compared with non-physically active controls with high vascular burden after controlling for individual Vascular Risk Factors (FWE *p* < 0.05)

|  |  |  |  | **MNI Coordinates of Local Maxima** | | | **WM Tracts** | |
| --- | --- | --- | --- | --- | --- | --- | --- | --- |
| **Clusters** | **Voxels** | **Value** | ***p*-value** | **X** | **Y** | **Z** | **JHU-ICBM-DTI-81 WM Labels** | **JHU-WM Tractography Atlas** |
| 4 | 2696 | 5 | **0.007** | 27 | -30 | 17 | Right Retrolenticular Part of Internal Capsule |  |
|  | 2696 | 4.73 | **0.007** | 50 | 0 | -12 | Unclassified | Right Inferior Longitudinal Fasciculus |
|  | 2696 | 4.53 | **0.007** | 27 | -26 | 15 | Right Retrolenticular Part of Internal Capsule |  |
|  | 2696 | 4.41 | **0.007** | 27 | -29 | 23 | Right Posterior Corona Radiata |  |
|  | 2696 | 4.29 | **0.007** | 33 | -30 | 8 | Right Retrolenticular Part of Internal Capsule | Right Inferior Fronto-Occipital Fasciculus |
|  | 2696 | 4.28 | **0.007** | 27 | -28 | 18 | Right Retrolenticular Part of Internal Capsule |  |
| 3 | 2077 | 4.51 | **0.008** | -22 | -8 | 14 | Left Posterior Limb of Internal Capsule |  |
|  | 2077 | 4.48 | **0.008** | -29 | -33 | 13 | Left Retrolenticular Part of Internal Capsule |  |
|  | 2077 | 4.48 | **0.008** | -21 | -10 | 10 | Left Posterior Limb of Internal Capsule |  |
|  | 2077 | 4.33 | **0.008** | -28 | -18 | 15 | Left External Capsule |  |
|  | 2077 | 4.31 | **0.008** | -28 | -16 | 19 | Left Superior Corona Radiata | Left Corticospinal Tract |
|  | 2077 | 4.28 | **0.008** | -28 | -18 | 17 | Left External Capsule | Left Corticospinal Tract |
| 2 | 286 | 4.95 | **0.012** | -8 | -18 | -2 | Unclassified | Left Anterior Thalamic Radiation |
|  | 286 | 4.44 | **0.012** | -13 | -21 | -2 | Unclassified | Left Anterior Thalamic Radiation |
|  | 286 | 4.41 | **0.012** | -10 | -25 | 5 | Unclassified | Left Anterior Thalamic Radiation |
|  | 286 | 4.39 | **0.012** | -9 | -24 | 8 | Unclassified | Left Anterior Thalamic Radiation |
|  | 286 | 4.06 | **0.012** | -8 | -14 | -1 | Unclassified | Left Anterior Thalamic Radiation |
|  | 286 | 4.02 | **0.012** | -6 | -11 | -1 | Unclassified | Left Anterior Thalamic Radiation |

Only tracts with > 100 voxels have been listed, Covariates: Age, body-mass index, diabetes, site of recruitment, smoking status, systolic blood pressure, total intracranial volume (TIV) and years of education
